# Supplementary material for: Impact of integrated care on trends in the rate of emergency department visits among older persons in Stockholm County: an interrupted time series analysis
Source: BMJ Open. 2020 Jun 3;10(6):e036182. doi: 10.1136/bmjopen-2019-036182 (PMC7279653; doi:10.1136/bmjopen-2019-036182)
Supplement: Supplementary data [file bmjopen-2019-036182supp001.pdf]

Supplementary Data

Table S1: Socio-demographic description of the inhabitants of Norrtälje in proportions (%) and means from 2000-2015

	**Year**	**2000**	**2001**	**2002**	**2003**	**2004**	**2005**	**2006**	**2007**	**2008**	**2009**	**2010**	**2011**	**2012**	**2013**	**2014**	**2015**
	N=	10,249	10,460	10,660	10,801	10,921	11,114	11,330	11,704	12,242	12,832	13,103	13,893	14,580	15,039	15,481	16,011
**Variables**		(%)	(%)	(%)	(%)	(%)	(%)	(%)	(%)	(%)	(%)	(%)	(%)	(%)	(%)	(%)	(%)
**Sex**	Male	45.29	45.38	45.54	45.72	45.83	46.07	46.44	46.69	46.80	47.11	47.15	47.47	47.79	48.05	48.08	47.98
	Female	54.71	54.62	54.46	54.28	54.17	53.93	53.56	53.31	53.20	52.89	52.85	52.53	52.21	51.95	51.92	52.02
**Age**	Mean in years	75.69	75.7	75.71	75.71	75.69	75.66	75.56	75.43	75.19	74.98	74.75	74.71	74.57	74.61	74.69	74.78
**Age groups**	65–79 years	74.82	73.85	72.74	72.49	72.53	72.70	72.44	73.11	73.89	74.49	75.74	75.83	76.88	77.12	77.24	77.70
	80+ years	25.18	26.15	27.26	27.51	27.47	27.30	27.56	26.89	26.11	25.51	24.26	24.17	23.12	22.88	22.76	22.30
**Income**	Group 1 (low)	31.00	29.75	29.37	35.54	36.59	35.62	31.29	31.64	31.23	30.75	30.95	30.43	29.21	29.29	28.95	28.22
	Group 2	21.98	23.15	22.32	22.07	21.32	21.51	20.56	20.30	19.80	19.54	19.64	20.15	25.33	25.32	25.21	25.14
	Group 3	19.01	18.12	19.32	17.33	17.21	16.72	18.87	19.28	19.84	20.60	20.38	2.50	17.25	17.90	17.74	18.27
	Group 4	14.06	14.56	14.50	14.95	14.74	15.25	16.92	17.06	16.76	16.97	16.96	34.38	15.49	15.07	15.35	15.30
	Group 5 (high)	9.02	9.71	9.55	9.99	10.03	10.80	12.10	11.44	12.18	11.86	11.77	12.27	11.98	11.69	11.99	12.39
	Missing	4.94	4.71	4.94	0.11	0.11	0.10	0.26	0.28	0.19	0.29	0.31	0.26	0.75	0.72	0.75	0.67
**Country of Birth**	Other	8.76	9.14	9.45	9.65	9.92	10.13	10.61	10.73	10.65	10.50	10.46	10.57	10.58	10.59	10.70	10.66
	Sweden	91.24	90.86	90.55	90.35	90.08	89.87	89.39	89.27	89.35	89.50	89.54	89.43	89.42	89.41	89.30	89.34
**Living situation**	Cohabitating	55.75	51.63	52.14	51.28	54.69	54.67	54.43	54.94	55.61	56.11	56.45	56.55	55.85	55.62	55.61	55.30
	Alone	44.25	43.30	43.02	43.74	45.31	45.33	45.57	45.06	44.39	43.89	43.55	43.45	44.15	44.38	44.39	44.70
	Missing		5.08	4.84	4.98												
	Died	4.84	4.58	4.84	4.98	4.77	4.87	4.57	4.37	4.23	4.11	4.25	4.05	3.61	3.78	3.40	3.49
**ED utilisation**	None	70.29	69.93	68.12	68.93	69.28	68.95	69.22	68.75	67.64	67.99	68.26	67.4	67.52	68.05	69.32	69.13
	1 visits	16.93	17.2	17.9	17.36	16.55	16.54	16.8	16.97	18.1	17.97	17.71	18.4	17.7	17.95	16.36	17.18
	2–3 visits	9.53	9.85	10.49	10.05	10.16	10.5	10.1	10.3	10.52	10.29	10.32	10.22	10.51	10.17	10.24	9.83
	4+ visits	3.25	3.02	3.49	3.67	4.01	4.01	3.87	3.98	3.74	3.76	3.72	3.97	4.27	3.83	4.08	3.85
**ED visits**	Mean no. visits	0.57	0.57	0.62	0.61	0.63	0.63	0.61	0.63	0.63	0.62	0.62	0.65	0.66	0.64	0.64	0.62

Table S2: Socio-demographic description of the inhabitants aged 65+ years of the rest of Stockholm County in proportions (%) and means from 2000-2015

	**Year**	**2000**	**2001**	**2002**	**2003**	**2004**	**2005**	**2006**	**2007**	**2008**	**2009**	**2010**	**2011**	**2012**	**2013**	**2014**	**2015**
	N=	261,608	262,922	262,398	263,688	265,654	268,125	272,192	278,409	286,508	296,660	307,293	316,813	326,014	334,981	343,047	349,958
**Variables**		(%)	(%)	(%)	(%)	(%)	(%)	(%)	(%)	(%)	(%)	(%)	(%)	(%)	(%)	(%)	(%)
**Sex**	Male	40.19	40.41	40.66	40.97	41.27	41.6	41.88	42.25	42.7	43.08	43.44	43.71	43.94	44.15	44.33	44.57
	Female	59.81	59.59	59.34	59.03	58.73	58.4	58.12	57.75	57.3	56.92	56.56	56.29	56.06	55.85	55.67	55.43
**Age**	Mean in years	76.1	76.14	76.18	76.16	76.12	76.09	75.99	75.81	75.58	75.35	75.17	75.02	74.92	74.86	74.88	74.92
**Age groups**	65–79 years	68.06	67.19	66.67	66.41	66.27	66.22	66.62	67.45	68.41	69.56	70.47	71.39	72.24	73.08	73.65	74.08
	80+ years	31.94	32.81	33.33	33.59	33.73	33.78	33.38	32.55	31.59	30.44	29.53	28.61	27.76	26.92	26.35	25.92
**Income**	Group 1 (low)	18.47	18.17	18.5	19.21	19.13	19.14	19.33	19.37	19.37	19.39	19.35	20.03	19.33	19.31	19.33	19.4
	Group 2	18.86	18.9	18.51	19.88	19.93	19.84	19.9	19.9	19.93	19.84	19.96	19.66	19.51	19.47	19.52	19.59
	Group 3	18.97	18.95	19.31	20	20.04	20.15	19.91	19.94	19.89	19.87	19.88	19.87	19.87	19.87	19.85	19.88
	Group 4	19.17	19.27	19.12	20.1	20.12	20.11	20.01	20	20.05	20.01	20.01	20.02	19.94	19.96	19.97	20.03
	Group 5 (high)	19.23	19.29	19.17	20.26	20.26	20.25	20.16	20.19	20.19	20.17	20.21	20.26	20.11	20.12	20.11	20.16
	Missing	5.31	5.43	5.4	0.56	0.52	0.5	0.69	0.6	0.58	0.71	0.6	0.16	1.24	1.26	1.22	0.95
**Country of Birth**	Other	16.53	17.06	17.6	18.06	18.53	18.96	19.35	19.41	19.47	19.48	19.5	19.76	20.09	20.4	20.75	21.19
	Sweden	83.47	82.94	82.4	81.94	81.47	81.04	80.65	80.59	80.53	80.52	80.5	80.24	79.91	79.6	79.25	78.81
**Living situation**	Cohabitating	48.66	45.41	45.76	45.84	48.9	48.93	48.97	49.25	49.64	50.01	50.39	50.7	49.91	50.22	50.38	50.53
	Alone	51.23	49.07	49.31	49.3	51	50.97	50.84	50.57	50.18	49.81	49.45	49.14	50.09	49.78	49.62	49.47
	Missing	0.12	5.53	4.93	4.86	0.1	0.11	0.19	0.19	0.18	0.18	0.16	0.16				
	Died	4.83	4.79	4.93	4.86	4.68	4.69	4.64	4.61	4.42	4.13	4.15	4.04	3.93	3.73	3.77	3.8
**ED utilisation**	None	71.02	70.75	71.29	71.35	72.36	72.1	69.02	67.99	69.02	70.62	70.63	70.24	70.77	71.02	71.55	70.11
	1 visits	15.98	16.41	16.12	16.3	16	16.32	17.66	17.58	17.23	16.7	16.68	16.73	16.57	16.52	16.3	17.22
	2–3 visits	9.77	9.71	9.52	9.38	8.91	8.9	10.13	10.65	10.18	9.56	9.55	9.75	9.52	9.4	9.22	9.55
	4+ visits	3.23	3.13	3.07	2.98	2.73	2.67	3.18	3.78	3.57	3.13	3.14	3.28	3.14	3.06	2.93	3.13
**ED visits**	Mean no. visits	0.56	0.56	0.55	0.54	0.52	0.51	0.59	0.64	0.61	0.56	0.56	0.58	0.56	0.55	0.54	0.57

Table S3: Description of ED care utilisation among stratified sub-groups in Norrtälje and the rest of Stockholm County.

	Norrtälje		Rest of Stockholm	
	**2000-06**	**2006-15**	**2000-06**	**2006-15**
**All inhabitants**				
**N=**	10,726	13,680.3	264,398.9	312,187.4
**Average no. per quarter**	1614.2	2137.7	33,037.5	39,043.95
**Average no. per quarter per person**	0.1503	0.1562	0.1250	0.1252
**Rate per quarter per 10,000 persons**	1503.5	1562.7	1249.9	1251.5
**Age adjusted rate per quarter per 10,000 persons**	1478.84	1584.17	1210.8	1262.3
**Males**				
**N=**	4,899.6	6,490	108,124.6	135,840.5
**Average no. per quarter**	798.1	1052.1	13,812.8	16,731.2
**Average no. per quarter per person**	0.163	0.162	0.128	0.125
**Rate per quarter per 10,000 persons**	1627.1	1620.4	1278.3	1252.3
**Age adjusted rate per quarter per 10,000 persons**	1674.5	1728.0	1308.7	1338.1
**Females**				
**N=**	5826.4	7,190.3	156,266.3	176.346.8
**Average no. per quarter**	816.1	1085.6	19224.7	22061.8
**Average no. per quarter per person**	0.14	0.15	0.12	0.13
**Rate per quarter per 10,000 persons**	1399.5	1510.6	1230.3	1251.8
**Age adjusted rate per quarter per 10,000 persons**	1336.6	1493.6	1135.2	1217.1
**65-79 years**				
**N=**	7427.7	9957.4	176,590.6	221,647
**Average no. per quarter**	905.2	1280.7	17880.2	21705.8
**Average no. per quarter per person**	0.12	0.13	0.10	0.098
**Rate per quarter per 10,000 persons**	1218.1	1287.5	1012.7	980.9
**Age adjusted rate per quarter per 10,000 persons**	822.7	891.5	681.7	681.8
**80+ years**				
**N=**	3,298.3	3,722.9	87,800.2	90,540.4
**Average no. per quarter**	708.9	857	15157.2	17330.2
**Average no. per quarter per person**	0.22	0.23	0.17	0.19
**Rate per quarter per 10,000 persons**	2145.8	2298.3	1727.0	1915.0
**Age adjusted rate per quarter per 10,000 persons**	660.05	700.57	529.3	580.4
**Lowest income**				
**N=**	3,536	4,060	49,630	60,637.3
**Average no. per quarter**	563.1	646.5	6843.5	7934.1
**Average no. per quarter per person**	0.158	0.159	0.14	0.13
**Rate per quarter per 10,000 persons**	1582.2	1593	1377.8	1307.4
**Age adjusted rate per quarter per 10,000 persons**	1449.6	1553.1	1261.9	1289.5
**Living alone**				
**N=**	4,744.1	6,061.2	132,660.9	155,606.1
**Average no. per quarter**	784.5	1071.2	17902.2	22492.0
**Average no. per quarter per person**	0.17	0.18	0.13	0.15
**Rate per quarter per 10,000 persons**	1650.3	1764.4	1349.2	1445.2
**Age adjusted rate per quarter per 10,000 persons**	1538.2	1676.6	1249.7	1360.9
**Born outside of Sweden**				
**N=**	1,026	1,451	47,216.8	62,437.7
**Average no. per quarter**	150.1	245.4	5679.6	7925.4
**Average no. per quarter per person**	0.15	0.17	0.12	0.13
**Rate per quarter per 10,000 persons**	1452.4	1683.0	1204.1	1268.3
**Age adjusted rate per quarter per 10,000 persons**	1591.5	1748.0	1250.0	1312.8

Supplementary figures corresponding to stratified analysis of socio-demographic sub-groups in Norrtälje municipality, the figures correspond to the estimates present in Table 1 of the main text.

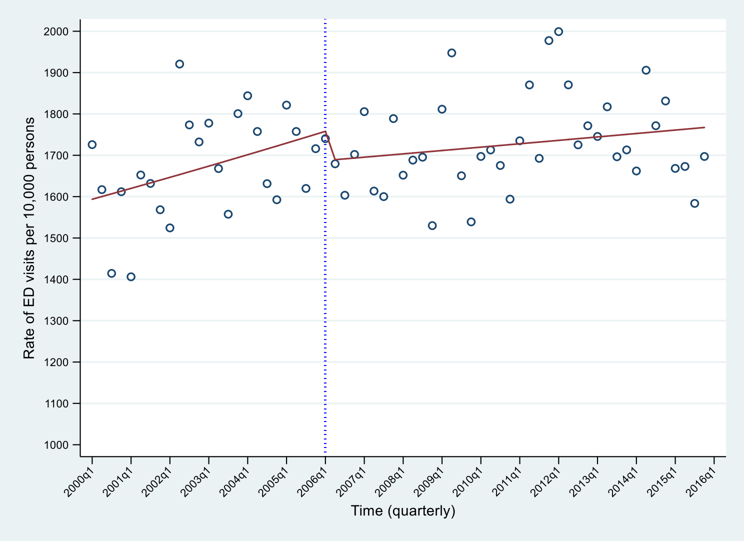

Figure S1: The trend in the rate of ED visits among male inhabitants in Norrtälje from 2000q1 to 2015q4. The circles represent the observed rate of ED visits per quarter per 10,000 persons, the vertical line represents the point where IC was implemented in Norrtälje. The solid lines represent the changes in the trend of the rate of ED visits in the pre-and post-intervention periods.

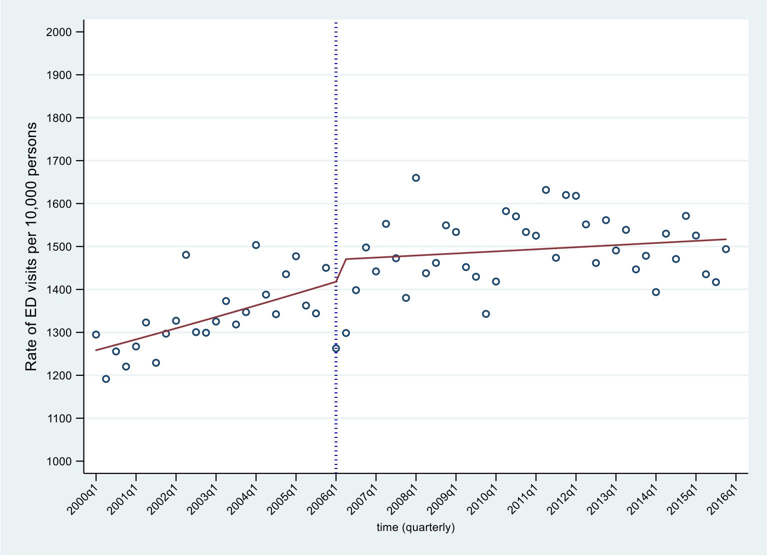

Figure S2: The trend in the rate of ED visits among female inhabitants in Norrtälje from 2000q1 to 2015q4. The circles represent the observed rate of ED visits per quarter per 10,000 persons, the vertical line represents the point where IC was implemented in Norrtälje. The solid lines represent the changes in the trend of the rate of ED visits in the pre-and post-intervention periods.

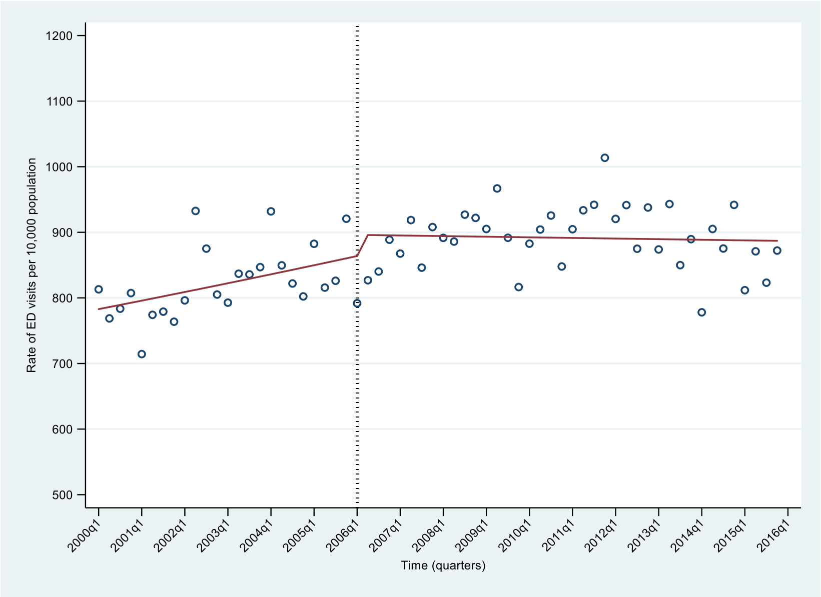

Figure S3: The trend in the rate of ED visits among all those 65-79 years in Norrtälje from 2000q1 to 2015q4. The circles represent the observed rate of ED visits per quarter per 10,000 persons, the vertical line represents the point where IC was implemented in Norrtälje. The solid lines represent the changes in the trend of the rate of ED visits in the pre-and post-intervention periods.

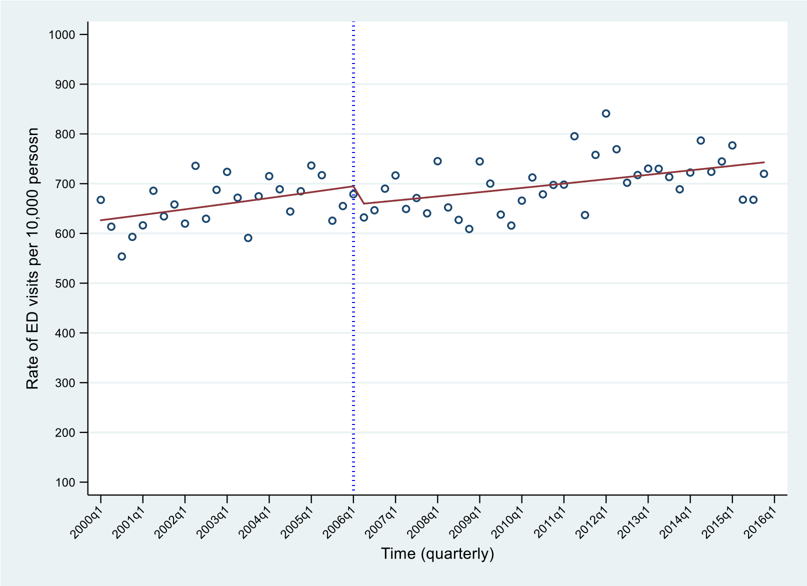

Figure S4: The trend in the rate of ED visits among all those 80+ years in Norrtälje from 2000q1 to 2015q4. The circles represent the observed rate of ED visits per quarter per 10,000 persons, the vertical line represents the point where IC was implemented in Norrtälje. The solid lines represent the changes in the trend of the rate of ED visits in the pre-and post-intervention periods.

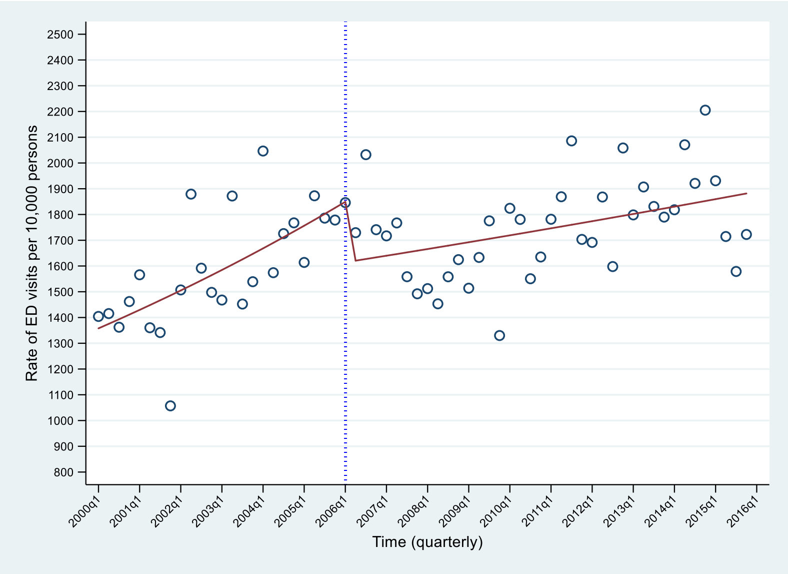

Figure S5: The trend in the rate of ED visits among all those born outside of Sweden living in Norrtälje from 2000q1 to 2015q4. The circles represent the observed rate of ED visits per quarter per 10,000 persons, the vertical line represents the point where IC was implemented in Norrtälje. The solid line represents the smooth trend of the rate of ED visits.

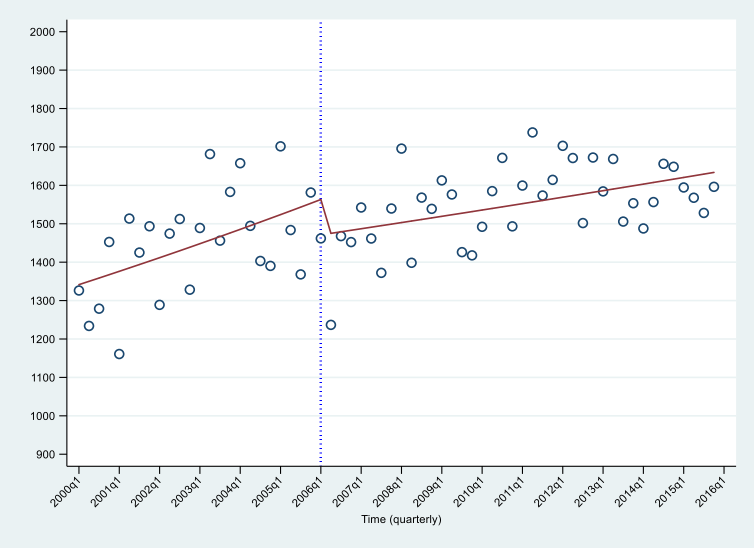

Figure S6: The trend in the rate of ED visits among all those in the lowest income group in Norrtälje from 2000q1 to 2015q4. The circles represent the observed rate of ED visits per quarter per 10,000 persons, the vertical line represents the point where IC was implemented in Norrtälje. The solid line represents the changes in the trend of the rate of ED visits in the pre-and post-intervention periods.

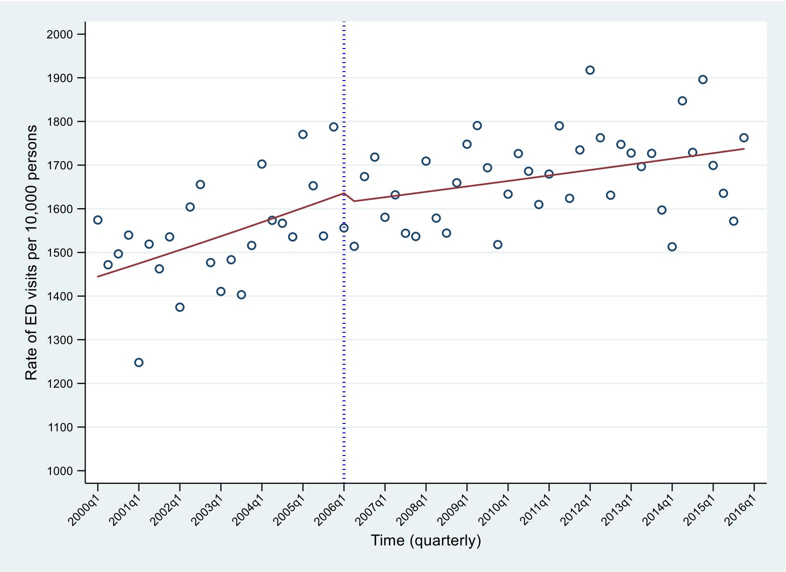

Figure S7: The trend in the rate of ED visits among all those living alone in Norrtälje from 2000q1 to 2015q4. The circles represent the observed rate of ED visits per quarter per 10,000 persons, the vertical line represents the point where IC was implemented in Norrtälje. The solid lines represent the changes in the trend of the rate of ED visits in the pre-and post-intervention periods.

Sensitivity Analysis

In the pre-intervention period 2000q1 to 2005q4 the trend of the rate of ED visits was increasing, following the implementation of IC, there was no significant level change (IRR: 0.971 CI 0.908 to 1.038), followed by a moderately decreasing change in the trend (IRR: O.999 CI: 0.996 to 1.01) in the post-intervention period relative to the pre-intervention period, however, this trend change was not significant. Though the changes in the rate of ED visits were not significant during the shortened study period 2000-2008. The trend follows the same direction as the observed in the main text figure 2, but the change was not as strong with the shorter follow-up period 2000-2008.

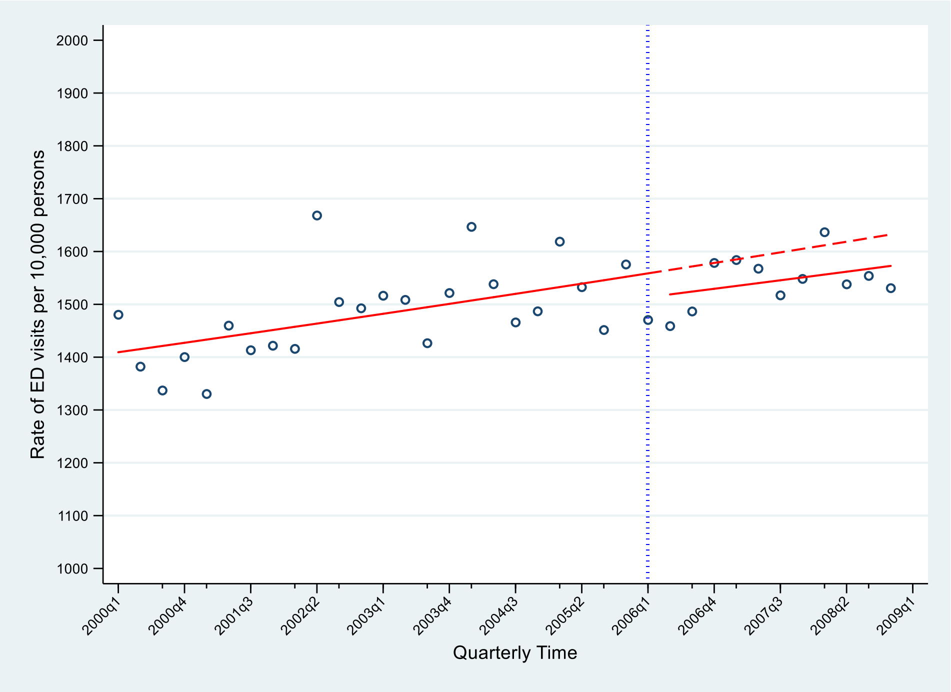

Figure S8: The trend in the rate of ED visits among all inhabitants 65+ years in Norrtälje from 2000q1 to 2008q4. The circles represent the observed rate of ED visits per 10,000 persons, the vertical line represents the implementation of integrated care (post-intervention trend from 2006q1) The solid line represents the temporal trend, and the step at the postintervention period is the estimated effect and the dashed red line represents the counterfactual trend if integrated care was not implemented in Norrtälje.
