# Supplementary material for: Impact of integrated care on trends in the rate of emergency department visits among older persons in Stockholm County: an interrupted time series analysis
Source: BMJ Open. 2020 Jun 3;10(6):e036182. doi: 10.1136/bmjopen-2019-036182 (PMC7279653; doi:10.1136/bmjopen-2019-036182)
Supplement: Author's manuscript [file bmjopen-2019-036182.draft_revisions.pdf]

BMJ Open

BMJ Open is committed to open peer review. As part of this commitment we make the peer review history of every article we publish publicly available.

When an article is published we post the peer reviewers' comments and the authors' responses online. We also post the versions of the paper that were used during peer review. These are the versions that the peer review comments apply to.

The versions of the paper that follow are the versions that were submitted during the peer review process. They are not the versions of record or the final published versions. They should not be cited or distributed as the published version of this manuscript.

BMJ Open is an open access journal and the full, final, typeset and author-corrected version of record of the manuscript is available on our site with no access controls, subscription charges or pay-per-view fees (http://bmjopen.bmj.com).

If you have any questions on BMJ Open's open peer review process please email info.bmjopen@bmj.com

BMJ Open

Impact of integrated care on the rate of emergency department visits among older persons in Stockholm County: An interrupted time series analysis

Journal:	*BMJ Open*
Manuscript ID	bmjopen-2019-036182
Article Type:	Original research
Date Submitted by the Author:	04-Dec-2019

Complete List of Authors:	Doheny, Megan; Karolinska Institute, Global Public Health<br>Agerholm, J; karolinska, Aging Research Center<br>Orsini, Nicola; Karolinska Institutet, Department of Public Health Sciences<br>Schön, Pär; Aging Research Center<br>Burström, Bo; Karolinska Institutet, Department of Public Health Sciences, Division of Social Medicine

Keywords:	Health policy < HEALTH SERVICES ADMINISTRATION & MANAGEMENT, Organisation of health services < HEALTH SERVICES ADMINISTRATION & MANAGEMENT, ACCIDENT & EMERGENCY MEDICINE

SCHOLARONE™
Manuscripts

I, the Submitting Author has the right to grant and does grant on behalf of all authors of the Work (as defined in the below author licence), an exclusive licence and/or a non-exclusive licence for contributions from authors who are: i) UK Crown employees; ii) where BMJ has agreed a CC-BY licence shall apply, and/or iii) in accordance with the terms applicable for US Federal Government officers or employees acting as part of their official duties; on a worldwide, perpetual, irrevocable, royalty-free basis to BMJ Publishing Group Ltd ("BMJ") its licensees and where the relevant Journal is co-owned by BMJ to the co-owners of the Journal, to publish the Work in this journal and any other BMJ products and to exploit all rights, as set out in our licence.

The Submitting Author accepts and understands that any supply made under these terms is made by BMJ to the Submitting Author unless you are acting as an employee on behalf of your employer or a postgraduate student of an affiliated institution which is paying any applicable article publishing charge ("APC") for Open Access articles. Where the Submitting Author wishes to make the Work available on an Open Access basis (and intends to pay the relevant APC), the terms of reuse of such Open Access shall be governed by a Creative Commons licence – details of these licences and which Creative Commons licence will apply to this Work are set out in our licence referred to above.

Other than as permitted in any relevant BMJ Author's Self Archiving Policies, I confirm this Work has not been accepted for publication elsewhere, is not being considered for publication elsewhere and does not duplicate material already published. I confirm all authors consent to publication of this Work and authorise the granting of this licence.

For peer review only

Title

Impact of Integrated Care on the rate of Emergency Department visits among older persons in Stockholm County: An interrupted time series analysis

Authors:

Megan Doheny

Department of Global Public Health, Karolinska Institutet, Stockholm, Sweden;

megan.doheny@ki.se

Janne Agerholm

Aging Research Center, Karolinska Institutet, Stockholm, Sweden

janne.agerholm@ki.se

Nicola Orsini

Department of Global Public Health, Karolinska Institutet, Stockholm, Sweden;

nicola.orsini@ki.se

Pär Schön

Aging Research Center, Karolinska Institutet, Stockholm, Sweden

par.schon@ki.se

Bo Burström

Department of Global Public Health, Karolinska Institutet, Stockholm, Sweden. Center for epidemiology and community medicine, Stockholm, County Council, Sweden.

bo.burstrom@ki.se

Corresponding auther:

Megan Doheny

e-mail: megan.doheny@ki.se

tel: +46 725486112

Address: Karolinska Institutet SE-171 77 Stockholm

For peer review only

For peer review only - http://bmjopen.bmj.com/site/about/guidelines.xhtml

Abstract

Objective
To investigate the association between the integrated care (IC) system implemented in Norrtälje municipality and the changes in the trends of emergency department (ED) visits.

Design
Interrupted time series analysis from 2000-2015.

Setting
Stockholm County

Participants
All inhabitants 65+ years in Stockholm County on the 31st of December of each study year.

Intervention
IC was established by combining the funding and administration of health and social care for the municipality of Norrtälje, within Stockholm County.

Outcome:
Rates of hospital-based emergency department visits

Results
IC was associated with a decrease in the rate of ED visits (IRR: 0.997 CI 0.995-0.998) among inhabitants 65+ years in Norrtälje. However, the rate of ED visits remained higher in Norrtälje than the rest of Stockholm in the pre-and post-intervention periods.Stratified analyses showed that IC was associated with a decrease in the trend of the rate of ED visits among both sexes, those 65-79 years, the lowest income group and born outside of Sweden. However, there was no significant decrease in the trend among those 80+ years.

Conclusion
The implementation of IC in Norrtälje was associated with a modest decrease in ED visits, though the rate of ED visits remained higher than in the rest of Stockholm. Changes in the composition of the population and contextual changes may have impacted our findings. Further research, using other outcome measures is needed to assess the impact of IC on health care utilisation.

Strengths and Limitations

- The study used a long time series of population-based data and could adjust for changes in composition of the population, using robust methods.
- The geographic setting of Norrtälje made it difficult to find an appropriate control community.
- Other contextual changes occurred during the study period which may have had an impact on results

For peer review only

Keywords: Integrated Care, Emergency Department visits, Time series analysis, Norrtälje Model

Word Count

Abstract 292 words
Main text 3,855 words

For peer review only - http://bmjopen.bmj.com/site/about/guidelines.xhtml

Introduction

The population of Sweden is rapidly ageing, those 80+ years represent the fastest growing group and are expected to double within 25 years (1). However, coupled with a longer life expectancy, there is an increased likelihood of older persons experiencing long-term health problems and functional disabilities associated with old age (2). Older persons often have complex health and social care needs due to multi-morbidity, functional and cognitive impairments and frailty, and as such are frequent users of health and social care services, often from multiple providers (3). Older persons can experience reduced quality of care and adverse health outcomes due to uncoordinated and fragmented delivery of care services care. Hence, it is important that the health and social care systems adapt to the needs of an ageing population, who would benefit from better alignment between and within health and social care services (4, 5).

In the Swedish health and social care system is highly decentralised. The responsibility of provision, management and financing of services is divided between three levels of governance. At the national level, the government sets out policy aims and directives, through legislation and economic incentives, while the 21 regions are responsible for the provision of health and medical care, and the 290 municipalities provide the social care services needed by older persons (6). Regions and municipalities are independent, as they both collect taxes to finance the majority of care services, set priorities and make decisions at the local and regional level (6). In addition, to the division of responsibility within the system, market-orientated reforms have been introduced to promote competition, freedom of choice and diversity of care providers, which has contributed to fragmentation in the Swedish care system (7, 8). Additionally, long-term care has been affected by a de-institutionalisation trend, with a reduction in the number of hospital beds and municipal institutional care. This has primarily been driven by cost containment but also the ageing in place policy, resulting in a growing number of older adults with health and social care needs living in the community (6, 9).

Hence, the Swedish health and social care system is fragmented, and those with complex health problems need to obtain the relevant information to make decisions about the care they should

For peer review only - http://bmjopen.bmj.com/site/about/guidelines.xhtml

seek and where to find it. This increases the risk for individuals to experience poor continuity and coordination of care between their various providers and may result in an increased use of emergency department (ED) care to meet their needs (10). The needs of those with complex health problems could be better met through coordinated care with long-term support, rather than in an acute and episodic care setting (11). The ED serves as an important link between the community primary health care services and the hospital based secondary and tertiary care (12). The staff in EDs are responsible for stabilising, diagnosing and treating patients, and moreover, they act as a gatekeeper determining whether an acute patient will be admitted into inpatient care or organising and negotiating their discharge into the community (13).

Integrated Care

Integrated care (IC), is an approach to organising and delivering care which is gaining in popularity as it could potentially provide care with greater efficiency and improve how patients receive and experience care (14). In theory, IC might be effective at overcoming fragmentation of care (14-18). The integration of care involves both vertical and horizontal processes. Vertical integration aims to bridge the gaps between different care services and providers that operate at different levels, i.e. from primary, secondary care to tertiary care. Horizontal integration refers to when different operating care organisations combine their efforts and resources to develop a seamless system for delivering care i.e. merging health and social care services and pooling financial resources.(11, 14, 16, 17.)

As health care systems are multi-layered and complex, establishing an IC system involves the consolidation within and between many different care providers (14, 16). IC can be considered within three levels: the macro-level i.e. the system, the meso-level i.e. organisation and the micro-level i.e. the clinical. The macro-level involves establishing structures to merge health and social care services across the continuum (15, 17, 18). The meso-level involves the re-organisation of care by focusing on sub-groups in the population that have greater needs. Finally, the micro-level focuses on the care required by individual users where care is coordinated and planned across the spectrum of providers and by involving the patient, their carers and families in the care decision to meet the goals of the patient (17). Better coordination and communication between professional groups is increasingly

For peer review only - http://bmjopen.bmj.com/site/about/guidelines.xhtml

important to address the complex needs of older persons, whose needs could be better fulfilled within an IC setting with coordination and continuity of care (4, 19).

Integrated Care – The Norrtälje Model

The Norrtälje model is an example of a comprehensive IC model in Sweden. The starting point was a threat of closing the hospital in Norrtälje municipality due to financial concerns, which led to a public appeal that gathered broad political support to save the hospital. In 2006, IC was initiated as a shared project between Norrtälje municipality and region Stockholm, with the objective of providing efficient, good quality and better coordinated care, while controlling costs (20, 21). The organisation of IC consists of a joint health and social care board with politicians from region Stockholm and Norrtälje municipality (21, 22). This board is responsible for the financial and organisation administration as well as the purchasing of care services from a jointly owned public company, Vårdbolaget TioHundra AB, and other private care providers. In Norrtälje, the IC initiative was a top-down process, which began in 2006, by creating a macro-structure focused on combining resources and shared structures, while further functional and clinical integration was rolled out in phases improving clinical integration and processes for sharing information, until 2016, when the project became permanent (see Figure 1) (21, 23).

Hence, our hypothesis was that the IC initiative in Norrtälje would facilitate better coordination of care and by doing so reduce the need for emergency department (ED) visits among the inhabitants 65+ years in Norrtälje. This study aimed to investigate the potential association between the implementation of an integrated care system in Norrtälje and the changes in the trends of ED visits in Norrtälje.

Methods

The study was designed with a quasi-experimental approach using repeated cross-sectional data, to assess the impact of integrated care on the utilisation of ED care by those 65+ years in Norrtälje (24). This study included all inhabitants 65+ years in Stockholm County on the 31st of December each year from 2000-2015. Those that moved out of Norrtälje or Stockholm County during a certain year were

For peer review only - http://bmjopen.bmj.com/site/about/guidelines.xhtml

excluded from that year. Exposure to IC was measured by being a registered inhabitant of Norrtälje municipality. An interrupted time series (ITS) analysis was used to compare the rates of ED visits per quarter in Norrtälje before and after the implementation of integrated care in January 2006 (25-27). Sub-group analyses were done to study trends in specific groups (by sex, age, socio-economic position, country of birth and living situation).

Data sources

We used repeated cross-sectional data from population-based registers covering the entire population of Stockholm County, obtaining data on health and health care utilisation from the region Stockholm administrative database (VAL) which contains information on all registered outpatient and inpatient care visits financed by region Stockholm. Data on ED visits were retrieved from the outpatient care register, and an ED visit was defined as an outpatient care visit to a hospital-based emergency department. Socio-demographic variables were obtained from the Longitudinal Integration Database for Social Insurance and Labour Market Studies (LISA), this database contains a collection of variables from various population registers linked individually through encrypted personal identity numbers. Sex was categorised into male and female. Age was calculated from the registered year of birth. Individual income was used as a measure of socio-economic position, grouped into categories based on the distribution of the net annual equalised household income and then ranked into quintiles. Country of birth was dichotomised into Sweden or other. Living situation was measured by civil status and was dichotomised into cohabiting or living alone.

Statistical analysis

An interrupted time series analysis with a generalised linear model was used to evaluate whether the post-intervention trends in the rates of ED visits differed significantly from the pre-intervention trends adjusting for the underlying trend and seasonality (26). The quarterly counts of ED visits were the dependent variable, the independent variables included in the model were a dummy variable distinguishing between the pre-intervention=0, and the post-intervention=1 period, and a variable counting the number of quarters that elapsed since the start of the study. This variable counting the

For peer review only - http://bmjopen.bmj.com/site/about/guidelines.xhtml

elapsed time allows us to estimate the underlying trend of the outcome. We hypothesised that there would be a gradual trend change due to the intervention, so an interaction term between elapsed time and the intervention was included in the model. Additionally, we included a dummy variable distinguishing the pre-and-post intervention period to estimate the level change immediately following the implementation of IC in Norrtälje. Plots of the residuals and the partial autocorrelation function of the residuals were assessed to ensure that the adjustment for seasonal effects and autocorrelation were adequate (25-27). The age-standardised rates of ED visits were used to adjust for the changes in the population over time. We had a total of 64 quarters as units of time in the study that were divided into two segments according to the intervention implementation. There were 25 quarters before, and 36 quarters after the implementation. Stratified analysis was performed to investigate differential effects among socio-demographic groups which could potentially benefit from an integrated care system including, those 80+ years, in lowest income group, living alone and born outside of Sweden. We assessed trends in the rate of ED visits among the inhabitants 65+ years and the relevant sub-groups in the rest of Stockholm County, to compare how the trends changed during the study period.

Results

In Norrtälje, the composition of the inhabitants 65+ years changed over time, the number of inhabitants increased from n=10,249 in 2000 to n=16,011 in 2015. The mean age decreased from 75.7 in 2000 to 74.8 years in 2015. Before the implementation of IC there was a larger proportion of females, while after the intervention the proportion of males 65+ increased by 3%, In Norrtälje, the proportion of the inhabitants 65+ in the lowest income group decreased from 31% to 28%, while the proportion of inhabitants born outside of Sweden increased from 8.8% to 10.7%, the proportion of persons living alone remained constant throughout the study period (see table S1). The inhabitants 65+ years in the rest of Stockholm County are described in table S2, generally they used lower levels of ED care compared to the inhabitants of Norrtälje, however, similarly to the inhabitants of Norrtälje the rate of ED visits increased from 1249.4 in the pre-intervention to 1251.2 per quarter per 10,000 persons in the post-intervention period (see table S3).

For peer review only - http://bmjopen.bmj.com/site/about/guidelines.xhtml

For peer review only

ITS analysis of the rate of ED visits among the inhabitants of Norrtälje

The relationship between the rate of ED visits and the implementation of integrated care among all inhabitants of Norrtälje 65+ years is demonstrated in Figure 2 and Table 1.There was an increasing trend in the rate of ED visits in the pre-intervention period. After adjusting for the underlying trends we observed no substantial level change in the rate of ED visits immediately after the post-intervention period compared to the pre-intervention period (IRR: 0.996 CI 0.971 to 1.022). However, there was a significant trend change in the rate of ED visits (IRR: 0.997 CI 0.995-0.998) in the post-intervention period compared to the pre-intervention period. Figure 2 demonstrates the counterfactual based on the pre-intervention trend estimates of the quarterly rate of ED visits, which shows that in the absence of IC the rate of visits in Norrtälje would have had a steeply increasing trend compared to the actual post-intervention trend.

The stratified analyses showed a significant decrease in level change in the rate of ED visits among males (IRR: 0.959 CI 0.937 to 0.983), immediately following the implementation of IC, but not among females (IRR: 1.036 CI 1.009 to 1.064). We observed a significant trend change in the rate of ED visits for both males (IRR: 0.997 CI 0.995 to 0.998) and females (IRR: 0.995 CI 0.994 to 0.998) in the post-intervention period compared with the preintervention period (see Figures S1-S2). There was a decrease in the level change observed among the inhabitants 80+ years (IRR: 0.946 CI 0.911 to 0.983) in the post-intervention compared to the pre-intervention, however, this level change was not observed among those 65-79 years (IRR: 1.009 CI 0.983 to 1.053). Furthermore, there was a significant decreasing trend change observed among those 65-79 years (IRR: 0.995 CI 0.993 to 0.997) in the post-intervention period compared to the pre-intervention period. No significant trend change in the rate of ED visits was observed among those 80+ years (IRR: 0.998 CI 0.997 to 1.001) in the post-intervention period compared to the pre-intervention period (Figure S3-S4).

There was a significant level change observed among those born outside of Sweden (IRR: 0.939 CI 0.987 to 0.996), however, this decrease was not sustained throughout the post-intervention period. Among those in the lowest income group we also found a significant level change (IRR: 0.916 CI 0.878 to 0.954) as well as a significant negative trend change in the post-intervention period. No

For peer review only - http://bmjopen.bmj.com/site/about/guidelines.xhtml

significant level change was observed among those inhabitants living alone in Norrtälje. However, the rate of ED visits was no longer increasing in the post-intervention period compared to the pre-intervention period (see Figures S5-S7).

ITS analysis of the rate of ED visits among the inhabitants 65+ in rest of Stockholm County

The trend in the rate of ED visits among the inhabitants of the rest of Stockholm County differed from those in Norrtälje municipality in the pre-intervention period and it was therefore not possible to do a comparative interrupted time serie analyses. The trends from the rest of the rregion Stockholm are depicted in figure 3. From 2000q1 to 2005q4 there was a downward trend in the rate of ED visits. There seems to be a slight increasing level change, however, the trend change in the post-intervention period is not significantly different from the trend in the pre-intervention period.

Discussion

During the study period the demographic composition and number of inhabitants 65+ years of Norrtälje changed. The utilisation of ED care increased slightly among inhabitants in Norrtälje from the start of the pre-intervention period to the end of the post-intervention period. Compared to the rest of Stockholm County the inhabitants of Norrtälje generally used more ED care. Our findings nevertheless suggest that the implementation of IC was associated with a decrease in the trend of ED visits in the post-intervention period compared to the pre-intervention. Although, we did not observe a reduction in the trend rate of ED visits immediately following the intervention. Our findings might be reflecting that the process of establishing the IC system was slow (from the macro, meso to the micro level), and that challenges were encountered during the process.

Our findings from the stratified analysis suggest that the rate of ED visits decreased among males, those 65-79 years, those in the lowest income group and those born outside of Sweden, after the introduction of IC. However, no significant change in the trend was observed among the inhabitants 80+ years.

The implementation of IC in Norrtälje was an initiative supported by local and regional authorities, however, throughout the post-intervention period additional nationwide policies were

For peer review only - http://bmjopen.bmj.com/site/about/guidelines.xhtml

introduced, which may have attenuated the effect of the intervention. The 2006 election led to a political shift, with changes in ambitions towards freedom of choice, competition and diversity. This resulted in a purchaser-provider split in Norrtälje, which was required in order to establish freedom of choice systems and competition in the health and social care. However, the fundamental ideas of IC were retained, i.e. integrating essential care services provided in older people's home. In Norrtälje they kept their own choice model in home-based care until 2010 when the choice reform in primary care was implemented nationally (7, 28, 29). As a result the TioHundra (IC provider) was no longer operating in isolation in Norrtälje. New providers were competing with the TioHundra which created an obstacle in the cohesion and collaboration needed between health and social care providers when developing IC (29), which may have impacted our findings.

Previous studies investigating the Norrtälje Model, have been very positive to the effects of the integration of health and social care in Norrtälje (21, 23).These previous evaluations have been predominantly process evaluations that involved interviewing stakeholders such as health care professionals, managers, politicians, and patients (22, 23). Due to the geographic location of Norrtälje, the hospital is the primary source of care in the area, so inhabitants may have a habit of going to the hospital when in need of care. Furthermore, the impetus for establishing IC was to save the local hospital. Thus, this care-seeking behaviour may persist even after the implementation of IC. These factors might contribute to explain the changes in the rate of ED visits we observed after the implementation of integrated care.

The implementation of IC in Norrtälje was initially supported by local and regional authorities, however, throughout the post-intervention period additional nationwide policies were introduced in 2009 and 2010, which may have attenuated the effect of the intervention. Choice reforms in social care in 2009 and in primary care in 2010, promoted the free establishment of, and competition between primary care clinics and home-help providers (7,28-30). New providers were competing with the Tiohundra which created an obstacle in the cohesion and collaboration needed between health and social care providers when developing integrated care which may have impacted our findings (21, 28, 29).

For peer review only - http://bmjopen.bmj.com/site/about/guidelines.xhtml

Previous research, investigating what impact IC could have on the use of ED care has shown mixed results (13,18). In Denmark, integrated care initiatives observed no effect on the use of ED care (31), similarly, in the UK, the results of a difference-in-difference study of Integrated Care Pilot studies reported no significant difference on the use ED services (32), while a study from Australia observed that integrated care reduced ED visits for persons 65+ years with a Chronic Obstructive Pulmonary Disease (COPD) or asthma diagnosis (33). Other research has indicated that certain features of integrated care such as case management and multi-disciplinary teams can be effective at reducing ED visits among older persons (4, 5, 34). However, within the Norrtälje model micro-level changes in the delivery of care were introduced gradually at a later stage of implementation, and we were unable to assess their effect on the rate of ED visits.

In Norrtälje, there was a top-down approach to implementing IC which started combining funding from the municipality of Norrtälje and the region Stockholm and having a shared management board for health and social care. However,further steps toward integration were gradual and encountered professional and technological hurdles (21, 23). There were differences in occupational cultures, professional boundaries and independent roles of health care professionals (23). This required further staff training and improvement in communication between and within the services, as there was no infrastructure for information sharing between health and social care practitioners. A shared information system was established in 2013 which enabled care practitioners to more easily coordinate the care across different services (21-23). Previous studies have indicated that financial factors are often barriers to achieving IC (35), and having shared funding is assumed to facilitate coordinated care. However, it has been reported that IC has not resulted in a sustained reduction in hospital use (15).

Strengths and Limitations

One advantage of this study was the use of population-based register data over a long time period. We could thereby take into account demographic changes in the composition of the population occurring during the study period. However, we had difficulty in finding an area that was demographically comparable, and followed a similar pre-intervention trend to Norrtälje which would have strengthened

For peer review only - http://bmjopen.bmj.com/site/about/guidelines.xhtml

our ability assess if the changes in the rate of visits we observed were due to the intervention or other factors (27). There are many potential explanations for this limitation, in Norrtälje. the hospital has a salient role in providing care in the community and is complemented by a few primary care clinics, as such there is less care availability from other sources in Norrtälje than in other parts of region Stockholm (21). The generalisability of these results might be limited as the geographic area and the community of Norrtälje are unique within region Stockholm.

Furthermore, an interrupted time series is most effective when there is a clear-cut change at a specific point in time that is expected to have an immediate effect (25-27). For this study, although the macro-structure facilitating IC was established in 2006, this was a complex intervention which took place at many levels and as such the implementation was gradual.

Our outcome is the aggregated number of ED visits per point in time, however, we cannot measure the reasons for the visits. Therefore, we cannot distinguish if there were more ED visits because of poor care coordination or more acute conditions. Furthermore, due to the role that ED care plays in the health care system there might be some floor effects in the rate of ED visits, as there will always be some need for ED care.

The quasi-experimental nature of this study is both a strength and a limitation (24). This approach is a strength as we were able to assess the impact of this large-scale shift to IC from a macro perspective, which gave us the possibility to follow the population before and after the intervention. Furthermore, having comprehensive datasets made it possible to study the total population and identify sub-groups which might benefited from IC. The downside to the quasi-experimental approach is our inability to isolate Norrtälje from external influences (24), as we have a long follow-up which coincided with other health and social care reforms which could influence our outcome. Finally, within this study we have no measures of how IC changed the inhabitants experienced care, whether the patients perceived that they received better care or were able to obtain the right combination of health and social care services to meet their individual needs. Previous research has observed that healthcare professionals working in an integrated care system perceive the quality of their provided care to be better (23, 29).

For peer review only - http://bmjopen.bmj.com/site/about/guidelines.xhtml

Conclusion

In summary, we found that the establishment of an integrated care initiative in Norrtälje was associated with a decrease in the trend of ED visits among the inhabitants of Norrtälje, though the changes observed were modest. Due to gradual implementation of different aspects of IC (e.g. staff training and shared information system) along with other policy changes in health and social care in the post-intervention period, it was difficult to disentangle the effect of the IC from the other policy changes. Further research is needed to to better understand what impact integrated care could have on the utilisation of other health and social care services.

For peer review only

Abbreviations

Emergency Department (ED), Integrated Care (IC), Primary Health Care (PHC), Generalised Linear Model (GLM), Confidence Interval (CI), Incidence Rate Ratio (IRR), Chronic Obstructive Pulmonary Disease (COPD)

Acknowledgements

A special thanks to the Equity and Health Policy research group at the department of Public Health Sciences at Karolinska Institutet and the Aging Research Center for helpful comments and suggestion of this paper.

Author contributions

MD, JA, NO, PS and BB conceived of the idea for, and participated in the design and the coordination this study. MD performed the statistical analysis and drafted the manuscript. MD, JA, NO, PS and BB were all involved in the interpretation of results and the revisions to the manuscript drafts. All authors read and approved the manuscript.

Funding

The research leading to these results was carried out as part of the Social Inequalities in Ageing (SIA) project, funded by NordForsk, project no.74637 and the Integrated Care Project funded by the Swedish Research Council for Health, Working Life and Welfare (Forte) project no 2017-02155.The funders of this research have had no direct role in the design, data collection, analysis or interpretation of data and in the writing of the manuscript.

Ethical approvals

Ethical approval for this study was obtained from the Regional Ethical Review Board in Stockholm, Sweden Dnr: 2016/299–31.

Participants consent

Individual consent to participate in this study was waived as this was a population-based study using administrative data.

Data statement

The data used will not be shared, as the materials are based on individual data which cannot be shared in accordance with Swedish law and GDPR.

Competing Interests

The authors declare no competing interests.

References

1. Statistics Sweden: Largest population increase expected among the elderly (2018). https://www.scb.se/en/finding-statistics/statistics-by-subject-area/population/population-projections/population-projections/pong/statistical-news/the-future-population-of-sweden-2018-2070/

For peer review only - http://bmjopen.bmj.com/site/about/guidelines.xhtml

2. WHO, World report on ageing and health: GenevaÖ Workd Health Organisation. 2015.
3. Meinow, B., M.G. Parker, and M. Thorslund, Complex health problems and mortality among the oldest old in Sweden: decreased risk for men between 1992 and 2002. European Journal of Ageing, 2010. 7(2): p. 81-90.
4. Briggs AM, Valentijn PP, Thiyagarajan JA, Araujo de Carvalho I. Elements of integrated care approaches for older people: a review of reviews. BMJ Open. 2018;8(4):e021194
5. Goodwin N. How should integrated care address the challenge of people with complex health and social care needs? Emerging lessons from international case studies. Int J Integr Care, (2015); 15:12.
6. Fritzell J, Salen L, Nelson K, Palme J and Schön P. European Social Policy Network [ESPN[, Country Profile: Sweden. Chapter 2 Systems and policies affecting social outcomes, 2016,2943.
7. Burström, B. (2015). Sweden – recent changes in welfare state arrangements. *International Journal of Health Services, 45*(1), 87-104.
8. Burström, B., Burström, K., Nilsson, G., Tomson, G., Whitehead, M., & Winblad, U. (2017). Equity aspects of the Primary Health Care Choice Reform in Sweden–a scoping review. *International journal for equity in health, 16*(1), 29.
9. SBU, *Omhändertagande av äldre som inkommer akut till sjukhus – med fokus på sköra äldre. En systematisk litteraturöversikt*. 2013: Stockholm: Statens beredning för medicinsk utvärdering [Swedish Council on Health Technology Assessment].
10. Meinow B, Parker M, Thorslund M. Consumers of eldercare in Sweden: The semblance ofchoice. Social Science and Medicine 2011;73;18 p233-248.
11. Hujala A, Taskinen H RS. How to support integration to promote care for people with multimorbidity in Europe? . Berlin: ICARE-4EU; 2016.
12. McCusker, J., Karp, I., Cardin, S., Durand, P., & Morin, J. (2003). Determinants of emergency department visits by older adults: A systematic review. *Academic Emergency Medicine, 10,* 1362-1370.
13. Nugus P, Carroll K, Hewett DG, Short A, Forero R, Braithwaite J. Integrated care in the emergency department: A complex adaptive systems perspective. Social Science and Medicine. 2010 Dec;71(11):1997-2004.
14. Kodner DL., Spreeuwenberg C. (2002) Integrated care: meaning, logic, applications, and implications – a discussion paper, International Journal of Integrated Care, 2, 1-6.
15. Mason A, Goddard M, Weatherly H, Chalkley M. Integrating funds for health and social care: an evidence review. J Health Serv Res Policy. 2015;20(3):177-88.
16. Leutz W. (1999) Five laws for integrating medical and social services: Lessons from the United States and the United Kingdom, Millbank quarterly 77(1), 77-110.
17. Goddard M., Mason AR. (2017) Integrated Care: A Pill for All Ills? International Journal of Health Policy and Management 6(1), 1-3.
18. Singer, S. J., Burgers, J., Friedberg, M., Rosenthal, M. B., Leape, L. L., & Schneider, E. C. (2011). Defining and measuring integrated patient care: Promoting the next frontier in healthcare delivery. *Medical Care Research and Review*, *68*(1), 112-127.
19. Tricco AC, Antony J, Ivers NM, Ashoor HM, Khan PA, Blondal E, et al. Effectiveness of quality improvement strategies for coordination of care to reduce use of health care services: a systematic review and meta-analysis. CMAJ. 2014;186(15):E568-78.
20. National Board of Health and Welfare [NBHW]. (2012b) Tillståndet och utvecklingen inom hälso+ och sjukvård och socialtjänst lägerapport 2012. (Trends in health care and social services, status report 2012). Swedeish Socialstyrelsen.

For peer review only - http://bmjopen.bmj.com/site/about/guidelines.xhtml

21. Bäck MA, Calltorp J. The Norrtaelje model: a unique model for integrated health and social care in Sweden. Int J Integr Care. 2015;15:e016.
22. Schön, P., Hagman, L. & Wånell, S.E. (2012). Kan två bli en? TioHundraprojektets effekter på vård och omsorg för målgruppen de mest sjuka äldre. [Can two become one? Implementing an integrated health and social care organization for the oldest old with complex health problems] Rapport 2012:01. Stiftelsen Stockholms läns Äldrecentrum [Stockholm Gerontology Research Center].
23. Øvretveit J, Hansson J, Brommels M. An integrated health and social care organisation in Sweden: creation and structure of a unique local public health and social care system. Health Policy. 2010;97(2-3):113-21.
24. Craig P, Katikireddi SV, Leyland A, Popham F. Natural Experiments: An Overview of Methods, Approaches, and Contributions to Public Health Intervention Research. Annu Rev Public Health. 2017;38:39-56.
25. Linden A, Arbor A, Conducting interrupted time-series analysis for single- and multiple-group comparisons. The Stata Journal (2015) 15, Number 2, p 480–500.
26. Lopez Bernal J, Cummins S, A. G. Interrupted time series regression for the evaluation of public health interventions: a tutorial. International Journal of Epidemiology. 2017;10(1093):348-55.
27. Kontopantelis, E., Doran, T., Springate, D. A., Buchan, I., & Reeves, D. (2015). Regression based quasi-experimental approach when randomisation is not an option: interrupted time series analysis. *BMJ (Clinical research ed.)*, *350*, h2750. doi:10.1136/bmj.h2750
28. Ahgren B. Competition and integration in Swedish health care. Health Policy. 2010;96(2):91-7.
29. Ström Falk R, Strano A & Schön P. Competing logics? The Coextistence of Integrated Care and Customer-choice in the Organisation and Provision of Home-based Care. 2015.
30. Leichsenring K, Rodrigues R, Winkelmann J, Falk R. Integrated Care, Competition and Choice - Removing barriers to care coordination in the context of market-oriented governance in Germany and Sweden. International Journal of Integrated Care. 2016;16(6):A89. DOI: http://doi.org/10.5334/ijic.2637
31. Schiøtz M, Price M, Frølich A, Søgaard J, Kristensen JK, Krasnik A, et al. Something is amiss in Denmark: a comparison of preventable hospitalisations and readmissions for chronic medical conditions in the Danish Healthcare system and Kaiser Permanente. BMC Health Serv Res. 2011;11:347.
32. RAND Europe, Ernst & Young LLP. National evaluation of the Departments of Health's Integrated Care Pilots: Final report. Cambridge. RAND Corporation, 2012.
33. Stephenson MD, Lisy K, Stern CJ, Feyer AM, Fisher L, Aromataris EC. The impact of integrated care for people with chronic conditions on hospital and emergency department utilization: a rapid review. Int J Evid Based Healthc. 2018.
34. Lemmens KM, Lemmens LC, Boom JH, Drewes HW, Meeuwissen JA, Steuten LM, et al. Chronic care management for patients with COPD: a critical review of available evidence. J Eval Clin Pract. 2013;19(5):734-52
35. Cameron A, Lart R, Bostock L, Coomber C. Factors that promote and hinder joint anintegrated working between health and social care services: a review of research literature. Health Soc Care Community. 2014;22(3):225-33.

For peer review only - http://bmjopen.bmj.com/site/about/guidelines.xhtml

Table 1: The trend changes in the rate of ED visits associated with the implementation of integrated care in Norrtälje between 2000-2015

	Rate of ED visits per quarter per 10,000 population		Age adjusted Rate of ED visits per quarter per 10,000 population		Level change (immediately following the intervention)		Trend change (after the intervention)	
	Before	After	Before	After	IRR (CI 95%)	p-value	IRR (CI 95%)	p-value
All inhabitants	1503.5	1562.7	1478.84	1584.17	0.996 (0.971 to 1.022)	0.793	0.997 (0.995 to 0.998)	0.000
Males	1627.0	1620.4	1674.5	1728.0	0.959 (0.937 to 0.983)	0.001	0.997 (0.995 to 0.998)	0.000
Females	1499.5	1510.6	1336.6	1493.6	1.036 (1.009 to 1.064)	0.008	0.995 (0.994 to 0.998)	0.000
65-79 years	1218.1	1287.5	822.7	891.5	1.037 (1.003 to 1.073)	0.034	0.995 (0.993 to 0.997)	0.000
80+ years	2145.8	2298.3	660.05	700.57	0.946 (0.911 to 0.983)	0.005	0.998 (0.996 to 1.00)	0.323
Lowest income	1582.2	1593.0	1449.6	1553.1	0.941 (0.917 to 0.965)	0.000	0.996 (0.994 to 0.997)	0.000
Living alone	1650.3	1764.4	1538.2	1676.6	0.987 (0.963 to 1.011)	0.301	0.996 (0.995 to 0.998)	0.000
Born outside of Sweden	1452.4	1683.0	1591.5	1748.0	0.872 (0.852 to 0.894)	0.000	0.991 (0.989 to 0.992)	0.000

- IRR incidence rate ratio, CI confidence interval
- The step change the level change immediately after the implementation of IC
- The trend change in the rate of ED visits in the post-intervention period

For peer review only - http://bmjopen.bmj.com/site/about/guidelines.xhtml

For peer review only

For peer review only - http://bmjopen.bmj.com/site/about/guidelines.xhtml

For peer review only

For peer review only - http://bmjopen.bmj.com/site/about/guidelines.xhtml

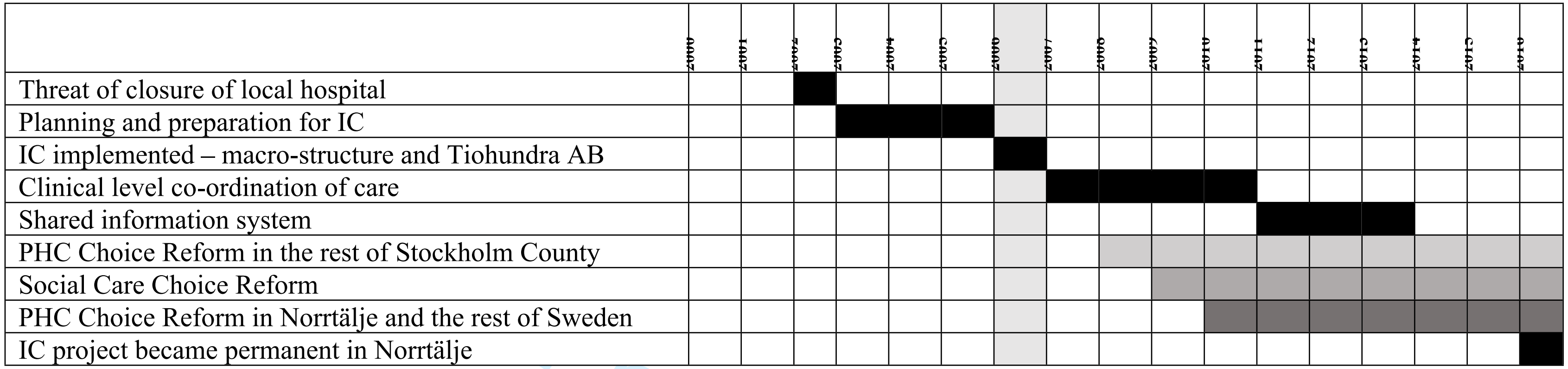

Figure 1: Overview of the timeline of the implementation of IC in Norrtälje

For peer review only

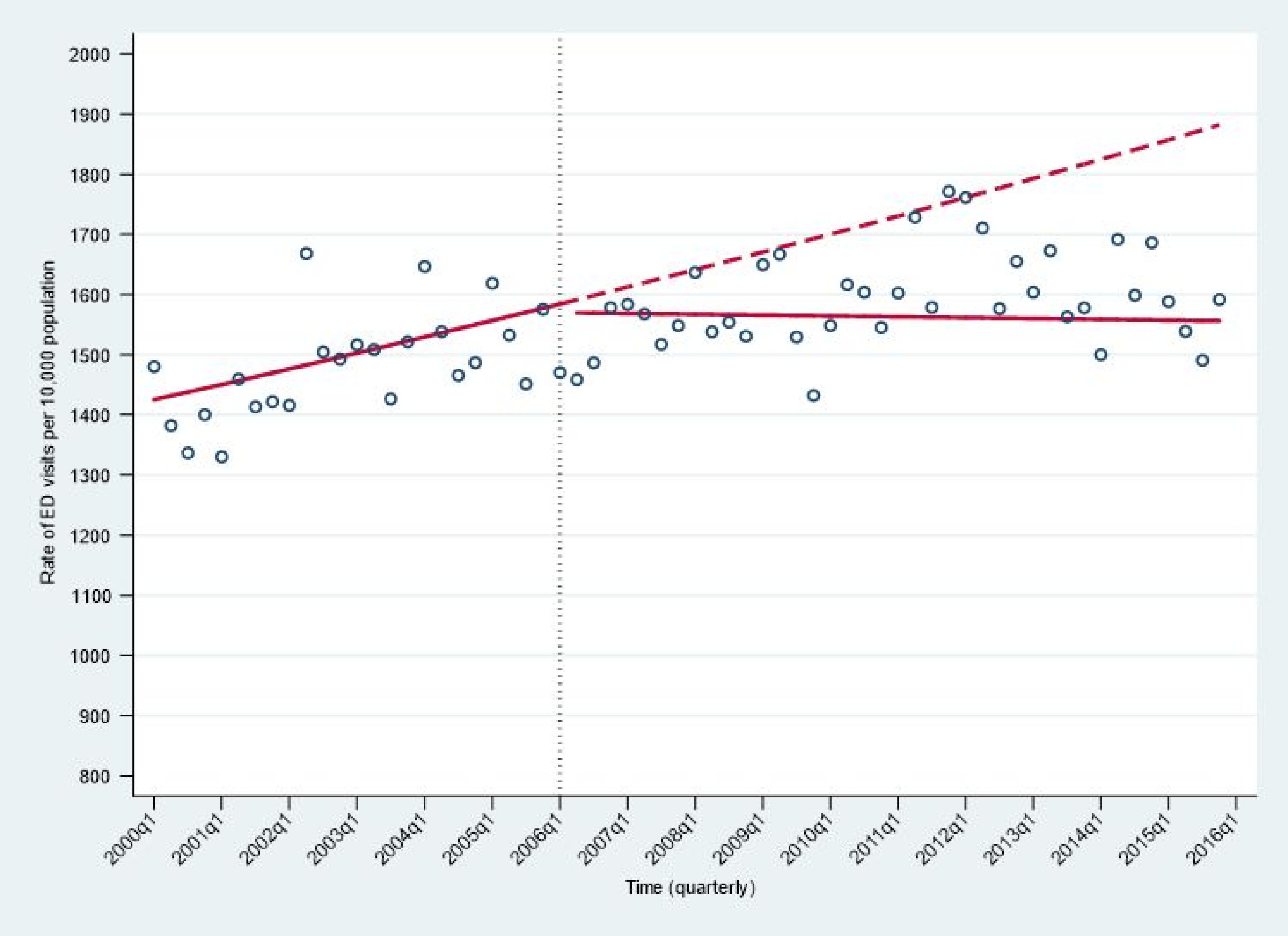

Figure 2: The trend in the rate of ED visits among all inhabitants 65+ years in Norrtälje from 2000q1 to 2015q4. The circles represent the observed rate of ED visits per 10,000 persons, the vertical line represents the implementation of integrated care (post-intervention trend from 2006q1) The solid line represents the temporal trend, and the step at the postintervention period is the estimated effect and the dashed red line represents the counterfactual trend if integrated care was not implemented in Norrtälje

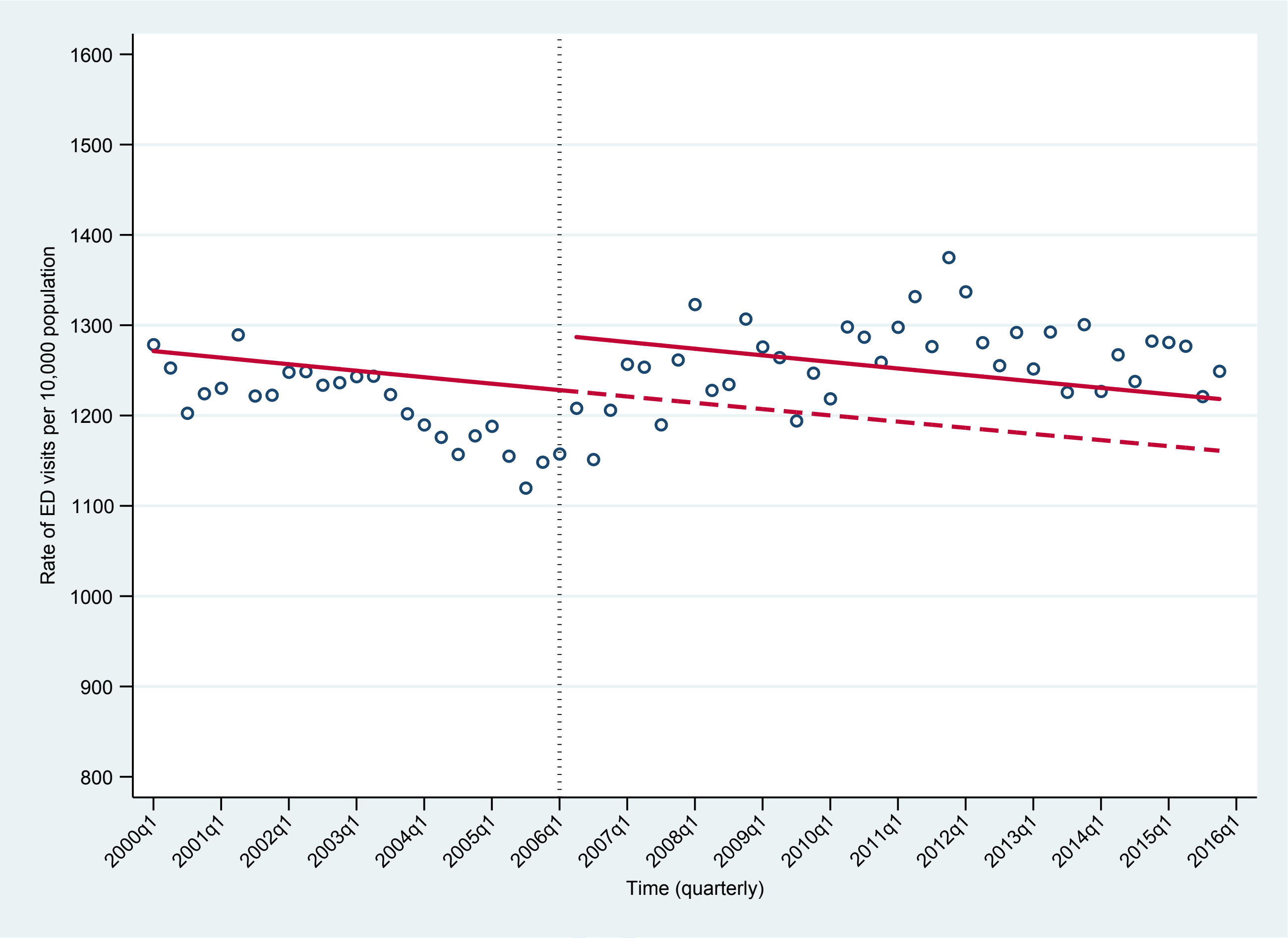

Figure 3: The trend in the rate of ED visits among the inhabitants 65+ years in the rest of Stockholm Countyfrom 2000q1 to 2015q4. The circles represent the observed rate of ED visits per 10,000 persons, the vertical line represents the point where IC was implemented in Norrtälje. The solid line represents the temporal trend, and the step at the post-intervention period is the estimated effect and the dashed red line represents the counterfactual trend if integrated care was not implemented in Norrtälje

review only

Supplementary Data

Table S1: Socio-demographic description of the inhabitants of Norrtälje in proportions (%) and means from 2000-2015

	**Year**	**2000**	**2001**	**2002**	**2003**	**2004**	**2005**	**2006**	**2007**	**2008**	**2009**	**2010**	**2011**	**2012**	**2013**	**2014**	**2015**
	N=	10,249	10,460	10,660	10,801	10,921	11,114	11,330	11,704	12,242	12,832	13,103	13,893	14,580	15,039	15,481	16,011
**Variables**		(%)	(%)	(%)	(%)	(%)	(%)	(%)	(%)	(%)	(%)	(%)	(%)	(%)	(%)	(%)	(%)
**Sex**	Male	45.29	45.38	45.54	45.72	45.83	46.07	46.44	46.69	46.80	47.11	47.15	47.47	47.79	48.05	48.08	47.98
	Female	54.71	54.62	54.46	54.28	54.17	53.93	53.56	53.31	53.20	52.89	52.85	52.53	52.21	51.95	51.92	52.02
**Age**	Mean in years	75.69	75.7	75.71	75.71	75.69	75.66	75.56	75.43	75.19	74.98	74.75	74.71	74.57	74.61	74.69	74.78
**Age groups**	65–79 years	74.82	73.85	72.74	72.49	72.53	72.70	72.44	73.11	73.89	74.49	75.74	75.83	76.88	77.12	77.24	77.70
	80+ years	25.18	26.15	27.26	27.51	27.47	27.30	27.56	26.89	26.11	25.51	24.26	24.17	23.12	22.88	22.76	22.30
**Income**	Group 1 (low)	31.00	29.75	29.37	35.54	36.59	35.62	31.29	31.64	31.23	30.75	30.95	30.43	29.21	29.29	28.95	28.22
	Group 2	21.98	23.15	22.32	22.07	21.32	21.51	20.56	20.30	19.80	19.54	19.64	20.15	25.33	25.32	25.21	25.14
	Group 3	19.01	18.12	19.32	17.33	17.21	16.72	18.87	19.28	19.84	20.60	20.38	2.50	17.25	17.90	17.74	18.27
	Group 4	14.06	14.56	14.50	14.95	14.74	15.25	16.92	17.06	16.76	16.97	16.96	34.38	15.49	15.07	15.35	15.30
	Group 5 (high)	9.02	9.71	9.55	9.99	10.03	10.80	12.10	11.44	12.18	11.86	11.77	12.27	11.98	11.69	11.99	12.39
	Missing	4.94	4.71	4.94	0.11	0.11	0.10	0.26	0.28	0.19	0.29	0.31	0.26	0.75	0.72	0.75	0.67
**Country of Birth**	Other	8.76	9.14	9.45	9.65	9.92	10.13	10.61	10.73	10.65	10.50	10.46	10.57	10.58	10.59	10.70	10.66
	Sweden	91.24	90.86	90.55	90.35	90.08	89.87	89.39	89.27	89.35	89.50	89.54	89.43	89.42	89.41	89.30	89.34
**Living situation**	Cohabitating	55.75	51.63	52.14	51.28	54.69	54.67	54.43	54.94	55.61	56.11	56.45	56.55	55.85	55.62	55.61	55.30
	Alone	44.25	43.30	43.02	43.74	45.31	45.33	45.57	45.06	44.39	43.89	43.55	43.45	44.15	44.38	44.39	44.70
	Missing		5.08	4.84	4.98												
	Died	4.84	4.58	4.84	4.98	4.77	4.87	4.57	4.37	4.23	4.11	4.25	4.05	3.61	3.78	3.40	3.49
**ED utilisation**	None	70.29	69.93	68.12	68.93	69.28	68.95	69.22	68.75	67.64	67.99	68.26	67.4	67.52	68.05	69.32	69.13
	1 visits	16.93	17.2	17.9	17.36	16.55	16.54	16.8	16.97	18.1	17.97	17.71	18.4	17.7	17.95	16.36	17.18
	2–3 visits	9.53	9.85	10.49	10.05	10.16	10.5	10.1	10.3	10.52	10.29	10.32	10.22	10.51	10.17	10.24	9.83
	4+ visits	3.25	3.02	3.49	3.67	4.01	4.01	3.87	3.98	3.74	3.76	3.72	3.97	4.27	3.83	4.08	3.85
**ED visits**	Mean no. visits	0.57	0.57	0.62	0.61	0.63	0.63	0.61	0.63	0.63	0.62	0.62	0.65	0.66	0.64	0.64	0.62

For peer review only

Table S2: Socio-demographic description of the inhabitants aged 65+ years of the rest of Stockholm County in proportions (%) and means from 2000-2015

	**Year**	**2000**	**2001**	**2002**	**2003**	**2004**	**2005**	**2006**	**2007**	**2008**	**2009**	**2010**	**2011**	**2012**	**2013**	**2014**	**2015**
	N=	261,608	262,922	262,398	263,688	265,654	268,125	272,192	278,409	286,508	296,660	307,293	316,813	326,014	334,981	343,047	349,958
**Variables**		(%)	(%)	(%)	(%)	(%)	(%)	(%)	(%)	(%)	(%)	(%)	(%)	(%)	(%)	(%)	(%)
**Sex**	Male	40.19	40.41	40.66	40.97	41.27	41.6	41.88	42.25	42.7	43.08	43.44	43.71	43.94	44.15	44.33	44.57
	Female	59.81	59.59	59.34	59.03	58.73	58.4	58.12	57.75	57.3	56.92	56.56	56.29	56.06	55.85	55.67	55.43
**Age**	Mean in years	76.1	76.14	76.18	76.16	76.12	76.09	75.99	75.81	75.58	75.35	75.17	75.02	74.92	74.86	74.88	74.92
**Age groups**	65–79 years	68.06	67.19	66.67	66.41	66.27	66.22	66.62	67.45	68.41	69.56	70.47	71.39	72.24	73.08	73.65	74.08
	80+ years	31.94	32.81	33.33	33.59	33.73	33.78	33.38	32.55	31.59	30.44	29.53	28.61	27.76	26.92	26.35	25.92
**Income**	Group 1 (low)	18.47	18.17	18.5	19.21	19.13	19.14	19.33	19.37	19.37	19.39	19.35	20.03	19.33	19.31	19.33	19.4
	Group 2	18.86	18.9	18.51	19.88	19.93	19.84	19.9	19.9	19.93	19.84	19.96	19.66	19.51	19.47	19.52	19.59
	Group 3	18.97	18.95	19.31	20	20.04	20.15	19.91	19.94	19.89	19.87	19.88	19.87	19.87	19.87	19.85	19.88
	Group 4	19.17	19.27	19.12	20.1	20.12	20.11	20.01	20	20.05	20.01	20.01	20.02	19.94	19.96	19.97	20.03
	Group 5 (high)	19.23	19.29	19.17	20.26	20.26	20.25	20.16	20.19	20.19	20.17	20.21	20.26	20.11	20.12	20.11	20.16
	Missing	5.31	5.43	5.4	0.56	0.52	0.5	0.69	0.6	0.58	0.71	0.6	0.16	1.24	1.26	1.22	0.95
**Country of Birth**	Other	16.53	17.06	17.6	18.06	18.53	18.96	19.35	19.41	19.47	19.48	19.5	19.76	20.09	20.4	20.75	21.19
	Sweden	83.47	82.94	82.4	81.94	81.47	81.04	80.65	80.59	80.53	80.52	80.5	80.24	79.91	79.6	79.25	78.81
**Living situation**	Cohabitating	48.66	45.41	45.76	45.84	48.9	48.93	48.97	49.25	49.64	50.01	50.39	50.7	49.91	50.22	50.38	50.53
	Alone	51.23	49.07	49.31	49.3	51	50.97	50.84	50.57	50.18	49.81	49.45	49.14	50.09	49.78	49.62	49.47
	Missing	0.12	5.53	4.93	4.86	0.1	0.11	0.19	0.19	0.18	0.18	0.16	0.16				
	Died	4.83	4.79	4.93	4.86	4.68	4.69	4.64	4.61	4.42	4.13	4.15	4.04	3.93	3.73	3.77	3.8
**ED utilisation**	None	71.02	70.75	71.29	71.35	72.36	72.1	69.02	67.99	69.02	70.62	70.63	70.24	70.77	71.02	71.55	70.11
	1 visits	15.98	16.41	16.12	16.3	16	16.32	17.66	17.58	17.23	16.7	16.68	16.73	16.57	16.52	16.3	17.22
	2–3 visits	9.77	9.71	9.52	9.38	8.91	8.9	10.13	10.65	10.18	9.56	9.55	9.75	9.52	9.4	9.22	9.55
	4+ visits	3.23	3.13	3.07	2.98	2.73	2.67	3.18	3.78	3.57	3.13	3.14	3.28	3.14	3.06	2.93	3.13
**ED visits**	Mean no. visits	0.56	0.56	0.55	0.54	0.52	0.51	0.59	0.64	0.61	0.56	0.56	0.58	0.56	0.55	0.54	0.57

For peer review only - http://bmjopen.bmj.com/site/about/guidelines.xhtml

Table S3: Description of ED care utilisation among stratified sub-groups in Norrtälje and the rest of Stockholm County.

	Norrtälje		Rest of Stockholm	
	**2000-06**	**2006-15**	**2000-06**	**2006-15**
**All inhabitants**				
**N=**	10,726	13,680.3	264,398.9	312,187.4
**Average no. per quarter**	1614.2	2137.7	33,037.5	39,043.95
**Average no. per quarter per person**	0.1503	0.1562	0.1250	0.1252
**Rate per quarter per 10,000 persons**	1503.5	1562.7	1249.9	1251.5
**Age adjusted rate per quarter per 10,000 persons**	1478.84	1584.17	1210.8	1262.3
**Males**				
**N=**	4,899.6	6,490	108,124.6	135,840.5
**Average no. per quarter**	798.1	1052.1	13,812.8	16,731.2
**Average no. per quarter per person**	0.163	0.162	0.128	0.125
**Rate per quarter per 10,000 persons**	1627.1	1620.4	1278.3	1252.3
**Age adjusted rate per quarter per 10,000 persons**	1674.5	1728.0	1308.7	1338.1
**Females**				
**N=**	5826.4	7,190.3	156,266.3	176.346.8
**Average no. per quarter**	816.1	1085.6	19224.7	22061.8
**Average no. per quarter per person**	0.14	0.15	0.12	0.13
**Rate per quarter per 10,000 persons**	1399.5	1510.6	1230.3	1251.8
**Age adjusted rate per quarter per 10,000 persons**	1336.6	1493.6	1135.2	1217.1
**65-79 years**				
**N=**	7427.7	9957.4	176,590.6	221,647
**Average no. per quarter**	905.2	1280.7	17880.2	21705.8
**Average no. per quarter per person**	0.12	0.13	0.10	0.098
**Rate per quarter per 10,000 persons**	1218.1	1287.5	1012.7	980.9
**Age adjusted rate per quarter per 10,000 persons**	822.7	891.5	681.7	681.8
**80+ years**				
**N=**	3,298.3	3,722.9	87,800.2	90,540.4
**Average no. per quarter**	708.9	857	15157.2	17330.2
**Average no. per quarter per person**	0.22	0.23	0.17	0.19
**Rate per quarter per 10,000 persons**	2145.8	2298.3	1727.0	1915.0
**Age adjusted rate per quarter per 10,000 persons**	660.05	700.57	529.3	580.4
**Lowest income**				
**N=**	3,536	4,060	49,630	60,637.3
**Average no. per quarter**	563.1	646.5	6843.5	7934.1
**Average no. per quarter per person**	0.158	0.159	0.14	0.13
**Rate per quarter per 10,000 persons**	1582.2	1593	1377.8	1307.4
**Age adjusted rate per quarter per 10,000 persons**	1449.6	1553.1	1261.9	1289.5
**Living alone**				
**N=**	4,744.1	6,061.2	132,660.9	155,606.1
**Average no. per quarter**	784.5	1071.2	17902.2	22492.0
**Average no. per quarter per person**	0.17	0.18	0.13	0.15
**Rate per quarter per 10,000 persons**	1650.3	1764.4	1349.2	1445.2
**Age adjusted rate per quarter per 10,000 persons**	1538.2	1676.6	1249.7	1360.9
**Born outside of Sweden**				
**N=**	1,026	1,451	47,216.8	62,437.7
**Average no. per quarter**	150.1	245.4	5679.6	7925.4
**Average no. per quarter per person**	0.15	0.17	0.12	0.13
**Rate per quarter per 10,000 persons**	1452.4	1683.0	1204.1	1268.3
**Age adjusted rate per quarter per 10,000 persons**	1591.5	1748.0	1250.0	1312.8

For peer review only - http://bmjopen.bmj.com/site/about/guidelines.xhtml

Supplementary figures corresponding to stratified analysis of socio-demographic sub-groups in Norrtälje municipality, the figures correspond to the estimates present in Table 1 of the main text.

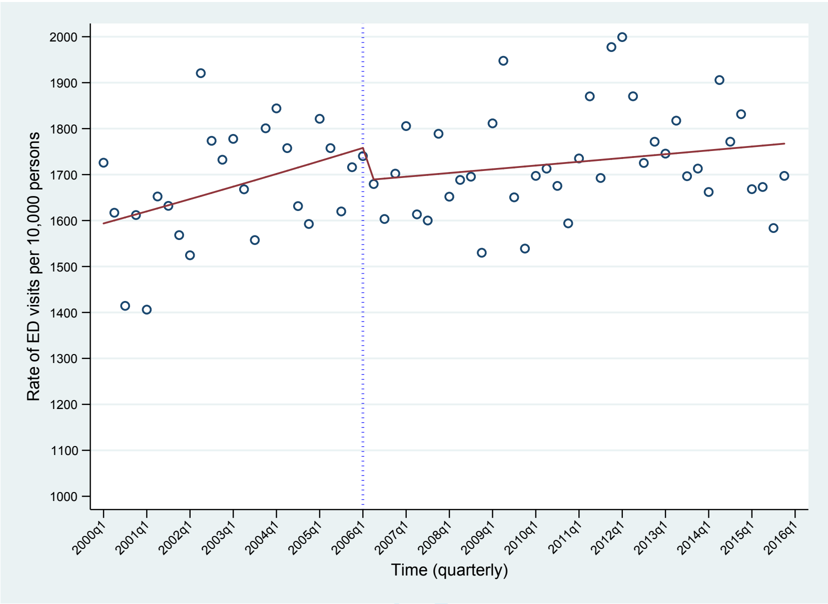

Figure S1: The trend in the rate of ED visits among male inhabitants in Norrtälje from 2000q1 to 2015q4. The circles represent the observed rate of ED visits per quarter per 10,000 persons, the vertical line represents the point where IC was implemented in Norrtälje. The solid lines represent the changes in the trend of the rate of ED visits in the pre-and post-intervention periods.

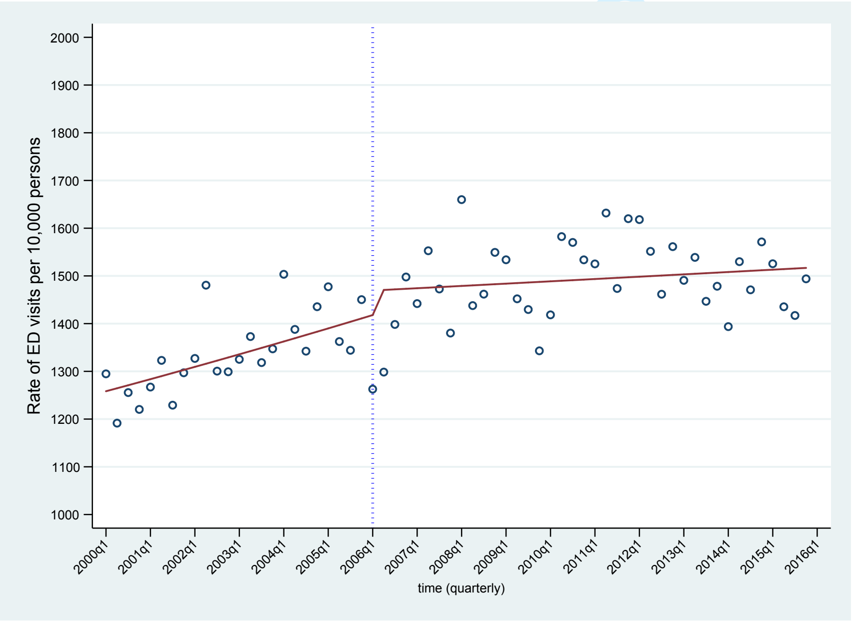

Figure S2: The trend in the rate of ED visits among female inhabitants in Norrtälje from 2000q1 to 2015q4. The circles represent the observed rate of ED visits per quarter per 10,000 persons, the vertical line represents the point where IC was implemented in Norrtälje. The solid lines represent the changes in the trend of the rate of ED visits in the pre-and post-intervention periods.

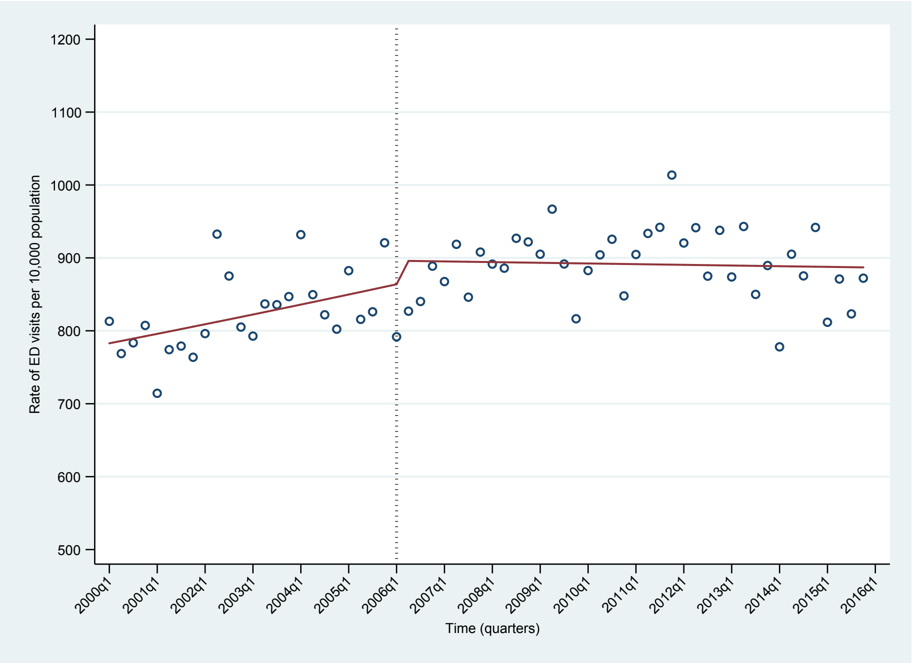

Figure S3: The trend in the rate of ED visits among all those 65-79 years in Norrtälje from 2000q1 to 2015q4. The circles represent the observed rate of ED visits per quarter per 10,000 persons, the vertical line represents the point where IC was implemented in Norrtälje. The solid lines represent the changes in the trend of the rate of ED visits in the pre-and post-intervention periods.

For peer review only - http://bmjopen.bmj.com/site/about/guidelines.xhtml

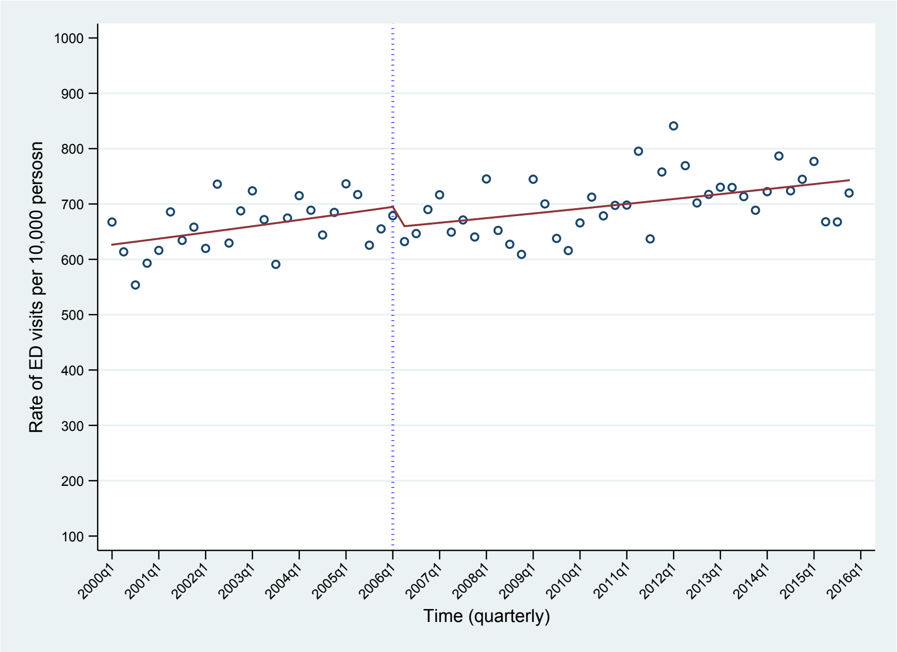

Figure S4: The trend in the rate of ED visits among all those 80+ years in Norrtälje from 2000q1 to 2015q4. The circles represent the observed rate of ED visits per quarter per 10,000 persons, the vertical line represents the point where IC was implemented in Norrtälje. The solid lines represent the changes in the trend of the rate of ED visits in the pre-and post-intervention periods.

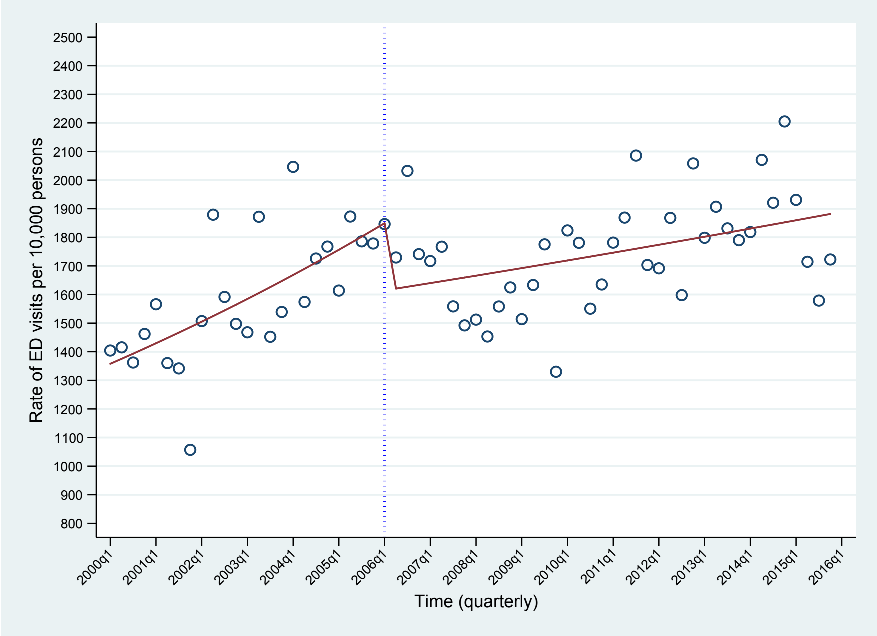

Figure S5: The trend in the rate of ED visits among all those born outside of Sweden living in Norrtälje from 2000q1 to 2015q4. The circles represent the observed rate of ED visits per quarter per 10,000 persons, the vertical line represents the point where IC was implemented in Norrtälje. The solid line represents the smooth trend of the rate of ED visits.

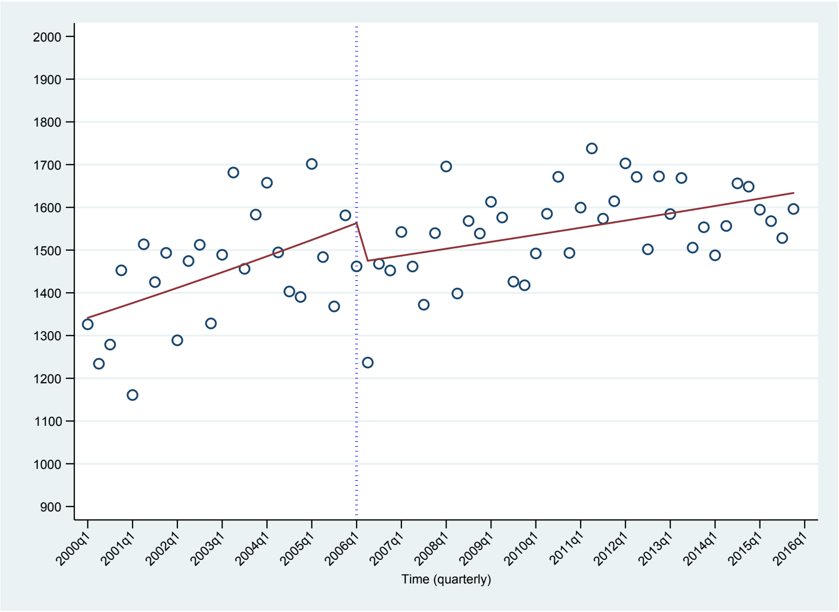

Figure S6: The trend in the rate of ED visits among all those in the lowest income group in Norrtälje from 2000q1 to 2015q4. The circles represent the observed rate of ED visits per quarter per 10,000 persons, the vertical line represents the point where IC was implemented in Norrtälje. The solid line represents the changes in the trend of the rate of ED visits in the pre-and post-intervention periods.

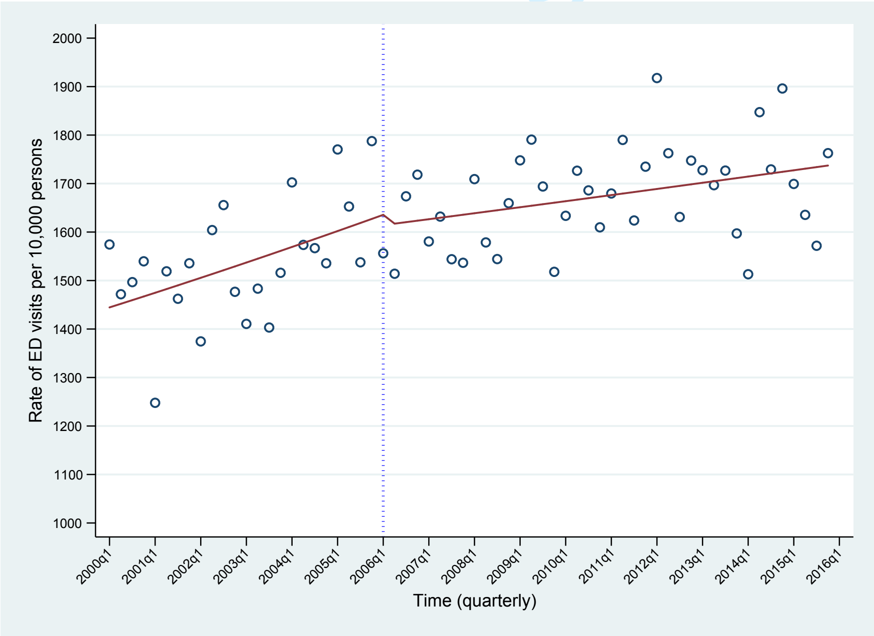

For peer review only - http://bmjopen.bmj.com/site/about/guidelines.xhtml

Figure S7: The trend in the rate of ED visits among all those living alone in Norrtälje from 2000q1 to 2015q4. The circles represent the observed rate of ED visits per quarter per 10,000 persons, the vertical line represents the point where IC was implemented in Norrtälje. The solid lines represent the changes in the trend of the rate of ED visits in the pre-and post-intervention periods.

For peer review only

BMJ Open

Impact of Integrated Care on trends in the rate of Emergency Department visits among older persons in Stockholm County: An interrupted time series analysis

Journal:	*BMJ Open*
Manuscript ID	bmjopen-2019-036182.R1
Article Type:	Original research
Date Submitted by the Author:	02-Mar-2020
Complete List of Authors:	Doheny, Megan; Karolinska Institute, Global Public Health<br>Agerholm, J; karolinska, Aging Research Center<br>Orsini, Nicola; Karolinska Institutet, Department of Public Health Sciences<br>Schön, Pär; Aging Research Center<br>Burström, Bo; Karolinska Institutet, Department of Public Health Sciences, Division of Social Medicine
**Primary Subject Heading**:	Health policy
Secondary Subject Heading:	Health services research, Patient-centred medicine
Keywords:	Health policy < HEALTH SERVICES ADMINISTRATION & MANAGEMENT, Organisation of health services < HEALTH SERVICES ADMINISTRATION & MANAGEMENT, ACCIDENT & EMERGENCY MEDICINE

SCHOLARONE™
Manuscripts

BMJ

I, the Submitting Author has the right to grant and does grant on behalf of all authors of the Work (as defined in the below author licence), an exclusive licence and/or a non-exclusive licence for contributions from authors who are: i) UK Crown employees; ii) where BMJ has agreed a CC-BY licence shall apply, and/or iii) in accordance with the terms applicable for US Federal Government officers or employees acting as part of their official duties; on a worldwide, perpetual, irrevocable, royalty-free basis to BMJ Publishing Group Ltd ("BMJ") its licensees and where the relevant Journal is co-owned by BMJ to the co-owners of the Journal, to publish the Work in this journal and any other BMJ products and to exploit all rights, as set out in our licence.

The Submitting Author accepts and understands that any supply made under these terms is made by BMJ to the Submitting Author unless you are acting as an employee on behalf of your employer or a postgraduate student of an affiliated institution which is paying any applicable article publishing charge ("APC") for Open Access articles. Where the Submitting Author wishes to make the Work available on an Open Access basis (and intends to pay the relevant APC), the terms of reuse of such Open Access shall be governed by a Creative Commons licence – details of these licences and which Creative Commons licence will apply to this Work are set out in our licence referred to above.

Other than as permitted in any relevant BMJ Author's Self Archiving Policies, I confirm this Work has not been accepted for publication elsewhere, is not being considered for publication elsewhere and does not duplicate material already published. I confirm all authors consent to publication of this Work and authorise the granting of this licence.

For peer review only

Title

Impact of Integrated Care on trends in the rate of Emergency Department visits among older persons in Stockholm County: An interrupted time series analysis

Authors:

Megan Doheny

Department of Global Public Health, Karolinska Institutet, Stockholm, Sweden;

megan.doheny@ki.se

Janne Agerholm

Aging Research Center, Karolinska Institutet, Stockholm, Sweden

janne.agerholm@ki.se

Nicola Orsini

Department of Global Public Health, Karolinska Institutet, Stockholm, Sweden;

nicola.orsini@ki.se

Pär Schön

Aging Research Center, Karolinska Institutet, Stockholm, Sweden

par.schon@ki.se

Bo Burström

Department of Global Public Health, Karolinska Institutet, Stockholm, Sweden. Center for Epidemiology and Community Medicine, Region Stockholm, Sweden.

bo.burstrom@ki.se

Corresponding auther:

Megan Doheny

e-mail: megan.doheny@ki.se

tel: +46 725486112

Address: Karolinska Institutet SE-171 77 Stockholm

For peer review only

For peer review only - http://bmjopen.bmj.com/site/about/guidelines.xhtml

Abstract

Objective

To investigate the association between the implementation of an integrated care (IC) system in Norrtälje municipality and changes in trends of the rate of emergency department (ED) visits.

Design

Interrupted time series analysis from 2000-2015.

Setting

Stockholm County

Participants

All inhabitants 65+ years in Stockholm County on the 31st of December of each study year.

Intervention

IC was established by combining the funding, administration and delivery of health and social care for older persons in Norrtälje municipality, within Stockholm County.

Outcome:

Rates of hospital-based emergency department visits

Results

IC was associated with a decrease in the rate of ED visits (IRR: 0.997 CI 0.995-0.998) among inhabitants 65+ years in Norrtälje. However, the rate of ED visits remained higher in Norrtälje than the rest of Stockholm in the pre-and post-intervention periods. Stratified analyses showed that IC was associated with a decline in the trend of the rate of ED visits among those 65-79 years, the lowest income group and born outside of Sweden. However, there was no significant decrease in the trend among those 80+ years.

Conclusion

The implementation of IC was associated with a modest change in the trend of ED visits in Norrtälje, though the rate of ED visits remained higher than in the rest of Stockholm. Changes in the composition of the population and contextual changes may have impacted our findings. Further research, using other outcome measures is needed to assess the impact of IC on health care utilisation.

Strengths and Limitations

- The study used a long time series of population-based data and could adjust for changes in composition of the population, using robust methods.
- The geographic setting of Norrtälje made it difficult to find an appropriate control community.
- Other contextual changes during the study period may have impacted the results

Keywords: Integrated Care, Emergency Department visits, Time series analysis, Norrtälje Model

Word Count

Abstract 296 words
Main text 3961 words

For peer review only - http://bmjopen.bmj.com/site/about/guidelines.xhtml

Introduction

The population of Sweden is rapidly ageing, those 80+ years represent the fastest growing group and are expected to double within 25 years (1). However, coupled with a longer life expectancy, there is an increased likelihood of older persons experiencing long-term health problems and functional disabilities associated with old age (2). Older persons often have complex health and social care needs due to multi-morbidity, functional and cognitive impairments and frailty, therefore, frequently need health and social care services from multiple providers (3). Older persons can experience reduced quality of care and adverse health outcomes due to uncoordinated and fragmented delivery of care services. Hence, it is important that the health and social care systems adapt to the needs of an ageing population, who could benefit from better alignment between and within health and social care services (4, 5).

The Swedish health and social care system is tax-funded and universal for all citizens, however, it is highly decentralised. The responsibility of provision, management and financing of services has three levels of governance. At the national level, the government sets policy, aims and directives, through legislation and economic incentives, while the 21 regions are responsible for the provision of health and medical care, and the 290 municipalities provide the social care services (i.e. home-help including both household and personal care services) needed by older persons (6). Regions and municipalities are independent, as they both collect taxes to finance the majority of care services, set priorities and make decisions at the regional and local level (6). Moreover, market-orientated reforms have been introduced in both health and social care which promote competition, freedom of choice and diversity of care providers, further contributing to fragmentation in the Swedish care system (7, 8). Furthermore, there has been a reduction in the number of municipal institutional care places and in the number of hospital beds, resulting in a change in how long-term care is provided. These trends have resulted in a growing number of older persons with complex care needs living in the community (6, 9).

Primary health care (PHC) is the basis of the Swedish health care system, where most patients with chronic diseases are treated, and includes home-health care services. PHC should also coordinate

For peer review only - http://bmjopen.bmj.com/site/about/guidelines.xhtml

with and be a link to social services for older persons. However, other specialist services may be required. The fragmentation in the Swedish system has placed those with complex care needs in a vulnerable position, as the patients must be able to obtain pertinent information relating to the care they need and which provider they should seek care from (8, 9). This increases the risk for individuals to experience poor continuity and coordination of care between multiple providers and may result in an increased use of emergency department (ED) care to meet their needs (10). The ED serves as an important link between PHC services and the hospital-based care (11). The staff in EDs are responsible for stabilising, diagnosing and treating patients, and moreover, they act as gatekeepers determining whether an acute patient will be admitted into inpatient care or organising and negotiating their discharge into the community (12). However, those with complex needs could be better met through coordinated care with long-term support, rather than in an acute and episodic care setting (13).

Integrated Care

Integrated care (IC), is an approach to organising and delivering care which could potentially provide care with greater efficiency and improve how patients receive and experience care (14). In theory, IC might be effective at overcoming fragmentation of care (14-18). The integration of care involves both vertical and horizontal processes. Vertical integration aims to bridge the gaps between different care services and providers that operate at different levels, i.e. from primary, secondary to tertiary care. Horizontal integration refers to when different care organisations combine their efforts and resources to develop a seamless system for delivering care i.e. merging health and social care services and pooling resources (13, 14, 16, 17.)

Health care systems are multi-layered and complex, establishing IC involves consolidation within and between many different care providers (14, 16). The implementation of IC can be considered within three levels: the macro-level i.e. the system, the meso-level i.e. organisation and the micro-level i.e. the clinical (15). The macro-level involves establishing structures to merge health and social care services across the continuum (15, 17, 18). The meso-level involves the re-organisation of care services targeting those with greater needs. Finally, the micro-level focuses on the care required by individual users, to facilitate the coordination of care across the spectrum of providers, by

For peer review only

involving the patient, their carers' and families' in the care decision to meet the goals of the patient (17). An IC setting might better meet the needs of older persons as IC promotes better coordination and communication between professional groups which should lead to better continuity of care (4, 19).

Integrated Care – The Norrtälje Model

The Norrtälje model is an example of an IC initiative in Sweden. Figure 1 provides an overview of the gradual implementation of IC in Norrtälje. The initiative began when the local hospital was under threat of closure due to financial concerns. This led to a public appeal that gathered broad political support to save the local hospital. In 2006, IC was initiated as a shared project between Norrtälje municipality and Region Stockholm. A joint health and social care board with politicians from Region Stockholm and Norrtälje municipality was formed, responsible for the financial and organisation administration as well as the purchasing of care services from a jointly owned public company, Vårdbolaget TioHundra AB, and other private care providers (20-22).

The Norrtälje model was set-up to provide care to the entire population of the municipality. The intervention had its base at the hospital and set out to join the efforts of health and social care services, through aligning medical documentation, care planning, rehabilitation, preventative care, home-help, home-health care and PHC services (20). Care teams were created with specific purposes to facilitate inter-professional group meetings, for training and to improve service delivery. The key to this approach was the requirement that all providers must operate within the integrated care chain. The model facilitated the development of a variety of care paths (e.g. dementia, stroke) (20), in addition to the national care plans for certain conditions already established in Sweden (23). The Norrtälje Model utilized a shared approach to policy and financing in order to promote a better integration of resources and care staff, and further, provided a stimulus for the development of a shared information system to facilitate IC (20, 22). Our hypothesis was that the IC initiative in Norrtälje would facilitate better coordination of care and by doing so reduce the utilisation of ED visits among the inhabitants 65+ years in Norrtälje. This study aimed to investigate the potential association between the implementation of an IC system and the changes in the trends of ED visits in Norrtälje.

For peer review only - http://bmjopen.bmj.com/site/about/guidelines.xhtml

Methods

The study was designed with a quasi-experimental approach using repeated cross-sectional data, to assess the impact of integrated care on the utilisation of ED care by those 65+ years in Norrtälje compared to the rest of Stockholm County (24). This study included all inhabitants 65+ years in Stockholm County on the 31st of December each year from 2000-2015. If an inhabitant died during the subsequent year, they were included in the study population for that year, if an inhabitant moved out of Stockholm Country during the study period they were excluded. Exposure to IC was measured by being a registered inhabitant of Norrtälje municipality. An interrupted time series (ITS) analysis was used to compare the rates of ED visits per quarter in Norrtälje before and after the implementation of integrated care in January 2006 (25-27). Sub-group analyses were done to study trends in specific groups (by sex, age, socio-economic position, country of birth and living situation).

Data sources

We used data from population-based registers covering the entire population of Stockholm County, obtaining data on health and health care utilisation from the Region Stockholm Healthcare Administrative Database (VAL) which contains information on all registered outpatient and inpatient care visits financed by Region Stockholm. Data on ED visits were retrieved from the outpatient care register, and an ED visit was defined as an outpatient care visit to a hospital-based emergency department.

Socio-demographic variables were obtained from the Longitudinal Integration Database for Social Insurance and Labour Market Studies (LISA), this database contains a collection of variables from various population registers linked individually through encrypted personal identity numbers. Sex was categorised into male and female. Age was calculated using the registered year of birth. Individual income was used as a measure of socio-economic position, grouped into categories based on the distribution of the net annual equivalized household income and then ranked into quintiles. Country of birth was dichotomised into Sweden or other. Living situation was measured by civil status and was dichotomised into cohabiting or living alone.

For peer review only - http://bmjopen.bmj.com/site/about/guidelines.xhtml

Patient and public involvement

The management group of Tiohundra AB were involved in the development of the research question and selection of the outcome measures based on the data available, they were not involved in the design and execution of the study. The data used in this study is based on encrypted personal identity number so that individuals in the study population are not identifiable. We have the possibility to disseminate our findings to the management group and the staff at Tiohundra AB.

Statistical analysis

An interrupted time series analysis with a generalised linear model with a Poisson distribution was used to evaluate whether the post-intervention trends in the rates of ED visits differed significantly from the pre-intervention trends adjusting for the underlying trend and seasonality (26). The quarterly counts of ED visits were the dependent variable, the independent variables included in the model were a dummy variable distinguishing between the pre-intervention=0, and the post-intervention=1 period, and a variable counting the number of quarters that elapsed since the start of the study. This variable counting the elapsed time allows us to estimate the underlying trend of the outcome. We hypothesised that there would be a gradual trend change due to the intervention, so an interaction term between elapsed time and the intervention was included in the model. Additionally, we included a dummy variable distinguishing the pre-and-post intervention period to estimate the level change immediately following the implementation of IC in Norrtälje. Plots of the residuals and the partial autocorrelation function of the residuals were assessed to ensure that the adjustment for seasonal effects and autocorrelation were adequate (25-27). The age-standardised rates of ED visits were used to adjust for the changes in the population over time. We had a total of 64 quarters as units of time in the study that were divided into two segments according to the intervention implementation. There were 25 quarters before, and 36 quarters after the implementation of IC. Stratified analysis was performed to investigate differential effects among socio-demographic groups which could potentially benefit from an IC system including, those 80+ years, in lowest income group, living alone and born outside of Sweden. We assessed trends in the rate of ED visits among the inhabitants 65+ years and the relevant sub-groups in the rest of Stockholm County, to compare how the trends changed during the study period.

For peer review only - http://bmjopen.bmj.com/site/about/guidelines.xhtml

Results

In Norrtälje, the composition of the inhabitants 65+ years changed over time, the number of inhabitants increased from n=10,249 in 2000 to n=16,011 in 2015. The mean age decreased from 75.7 in 2000 to 74.8 years in 2015. Before the implementation of IC there was a larger proportion of females, while after the intervention the proportion of males 65+ increased by 3%, The proportion of inhabitants 65+ in the lowest income group decreased from 31% to 28%, while the proportion born outside of Sweden increased from 8.8% to 10.7%, the proportion of persons living alone remained constant throughout the study period (see Table S1).

In contrast, the population in the rest of Stockholm County, had a mean age 76.1 years in 2000 and 74.9 years in 2015, there was a higher proportion of inhabitants that were female, in the highest income group, born outside of Sweden and living alone (see Table S2). The inhabitants 65+ years in the rest of Stockholm County generally used lower levels of ED care compared to the inhabitants of Norrtälje, however, similarly to the inhabitants of Norrtälje the rate of ED visits increased between 2000-2015 (see Table S3).

ITS analysis of the rate of ED visits among the inhabitants of Norrtälje

The relationship between the rate of ED visits and the implementation of integrated care among all inhabitants of Norrtälje 65+ years is demonstrated in Figure 2 and Table 1. There was an increasing trend in the rate of ED visits in the pre-intervention period. After adjusting for the underlying trends, we observed no substantial level change in the rate of ED visits (IRR: 0.996 CI 0.971 to 1.022). However, there was a significant trend change in the rate of ED visits (IRR: 0.997 CI 0.995-0.998) in the post-intervention period compared to the pre-intervention period. Figure 2 demonstrates the counterfactual trend based on the pre-intervention trend estimates of the quarterly rate of ED visits, which shows that in the absence of IC the rate of ED visits in Norrtälje would have had an increasing trend compared to the actual post-intervention trend.

The stratified analyses showed a significant level change in the rate of ED visits among males (IRR: 0.959 CI 0.937 to 0.983), immediately following the implementation of IC, but not among females (IRR: 1.036 CI 1.009 to 1.064). We observed a significant trend change in the rate of ED

For peer review only - http://bmjopen.bmj.com/site/about/guidelines.xhtml

visits for both males (IRR: 0.997 CI 0.995 to 0.998) and females (IRR: 0.995 CI 0.994 to 0.998) in the post-intervention period compared with the preintervention period (see Figures S1-S2). There was a decrease in the level of the rate of ED visits among those 80+ years (IRR: 0.946 CI 0.911 to 0.983), however, this level change was not observed among those 65-79 years (IRR: 1.009 CI 0.983 to 1.053). Furthermore, there was a significant decreasing trend change observed among those 65-79 years (IRR: 0.995 CI 0.993 to 0.997) in the post-intervention period compared to the pre-intervention period. However, there was no significant trend change in the rate of ED visits observed among those 80+ years (IRR: 0.998 CI 0.997 to 1.001) in the post-intervention period compared to the pre-intervention period (Figure S3-S4).

There was a significant level change observed among those born outside of Sweden (IRR: 0.939 CI 0.987 to 0.996), however, this decrease was not sustained throughout the post-intervention period. Among those in the lowest income group we also found a significant level change (IRR: 0.916 CI 0.878 to 0.954) as well as a significant trend change in the post-intervention period. No significant level change was observed among those inhabitants living alone in Norrtälje. However, the rate of ED visits was no longer increasing in the post-intervention period compared to the pre-intervention period (see Figures S5-S7). A sensitivity analysis was performed on all inhabitants 65+ years using a shorter timespan 2000-2008. The trend of the rate of ED visits followed the same direction, which was observed in Figure 2, however, the changes were not significant (see Figure S8).

ITS analysis of the rate of ED visits among the inhabitants 65+ in rest of Stockholm County

The trend in the rate of ED visits among the inhabitants of the rest of Stockholm County differed from those in Norrtälje municipality in the pre-intervention period and it was therefore not possible to do a comparative interrupted time series analysis. The trends from the rest of Stockholm County are depicted in Figure 3. From 2000q1 to 2005q4 there was a downward trend in the rate of ED visits. There was an increase in the level of ED visits, however, the trend between 2006q1 to 2015q4 was not significantly different from the trend in the pre-intervention period.

For peer review only

For peer review only - http://bmjopen.bmj.com/site/about/guidelines.xhtml

Discussion

Our findings suggest that the implementation of IC was associated with a decrease in the trend of ED visits in the post-intervention period compared to the pre-intervention among all those 65+ years. However, the utilisation of ED care increased slightly among inhabitants in Norrtälje from the start of the pre-intervention period to the end of the post-intervention period. Compared to the rest of Stockholm County the inhabitants of Norrtälje generally used more ED care. Additionally, the counterfactual demonstrated that the trend in the rate of ED visits would have increased if IC was not introduced. Our findings from the stratified analyses suggest that the rate of ED visits decreased among males, those 65-79 years, those in the lowest income group and those born outside of Sweden, after the introduction of IC. However, no significant change in the trend was observed among the inhabitants 80+ years. During the study period the demographic composition changed and number of inhabitants 65+ years increased in Norrtälje.

Previous studies investigating the Norrtälje Model, have been very positive to the effects of the integration of health and social care in Norrtälje (20, 22).These previous evaluations have been predominantly process evaluations interviewing stakeholders such as health care professionals, managers, politicians, and patients (21, 22). Due to the geographic location of Norrtälje, the hospital is the primary source of care in the area, so inhabitants may have a habit of going to the hospital when in need of care. Furthermore, the impetus for establishing IC was to save the local hospital. Thus, this care-seeking behaviour may persist even after the implementation of IC (22). These factors might contribute to explain the modest changes in the rate of ED visits we observed after the implementation of integrated care.

The implementation of IC in Norrtälje was an initiative supported by local and regional authorities. However, throughout the post-intervention period additional nationwide policies were introduced, which may have influenced the effect of the intervention. After 2006 there were changes in ambitions towards increasing freedom of choice, competition and diversity. This resulted in a purchaser-provider split in Norrtälje, which in turn, resulted in an increase of providers not part of IC, that competed with IC services (28 ,29). However, the fundamental ideas of IC were retained, i.e. integrating essential care services provided in older people's home (29 ,30). Norrtälje maintained its

For peer review only - http://bmjopen.bmj.com/site/about/guidelines.xhtml

own choice model in home-healthcare services until 2010 when the choice reform in primary care was implemented nationally (7, 28, 30). From 2010, the TioHundra (IC provider) was no longer the only main care provider in Norrtälje. New providers were competing with the TioHundra which created an obstacle in the cohesion and collaboration needed between health and social care providers when developing IC (29 ,30). This may have impacted our findings.

In Norrtälje, there was a top-down approach to implementing IC which started by combining funding from Norrtälje Municipality and Region Stockholm and through a shared management board. However, further steps toward integration were gradual and encountered professional and technological hurdles (20, 22). There were differences in occupational cultures, professional boundaries and rhe independent roles of health care professionals (22). This required further staff training and improvement in communication between and within the services, as there was no infrastructure for information sharing between health and social care practitioners. A shared information system was established in 2013 which enabled care practitioners to more easily coordinate the care across different services (20-22). Previous studies have indicated that financial factors are often barriers to achieving IC (31), and having shared funding is assumed to facilitate coordinated care. However, it has been reported that IC has not resulted in a sustained reduction in hospital use (15).

Previous research, investigating what impact IC could have on the use of ED care has shown mixed results (12, 18). In Denmark, studies of integrated care initiatives observed no effect on the use of ED care (32), similarly, in the UK, the results of a difference-in-difference study of Integrated Care Pilot studies reported no significant difference on the use ED services (33), while a study from Australia observed that integrated care reduced ED visits for persons 65+ years with a Chronic Obstructive Pulmonary Disease (COPD) or asthma diagnosis (34). Other research has indicated that certain features of integrated care such as case management and multi-disciplinary teams can be effective at reducing ED visits among older persons (4, 5, 35). However, within the Norrtälje model micro-level changes in the delivery of care were introduced gradually at a later stage of implementation.

For peer review only - http://bmjopen.bmj.com/site/about/guidelines.xhtml

Strengths and Limitations

One advantage of this study was the use of population-based register data over a long time period. We could thereby take into account demographic changes in the composition of the population occurring during the study period. However, we had difficulty in finding an area that was comparable and followed a similar pre-intervention trend to Norrtälje to strengthen our ability assess if the changes observed were due to the intervention or other factors (25, 27). There are many potential explanations for this limitation. In Norrtälje, the hospital has a salient role in providing care in the community and therefore, inhabitants use hospital-based care in a different way, as it is a rural area which does not have the same access to specialist care compared to those in the rest of Stockholm County. Therefore, the generalisability of these results might be limited.

Furthermore, an interrupted time series is most effective when there is a clear-cut change at a specific point in time that is expected to have an immediate effect (25). For this study, although the macro-structure facilitating IC was established in 2006, this was a complex intervention which took place at many levels and as such the implementation was gradual.

Our outcome is the aggregated number of ED visits per point in time, however, we cannot measure the reasons for the visits. Therefore, we cannot distinguish if there were more ED visits because of poor care coordination or more acute conditions. Furthermore, due to the role that ED care plays in the health care system there might be some floor effects in the rate of ED visits, as there will always be some need for ED care.

The quasi-experimental nature of this study is both a strength and a limitation (24). This approach is a strength as we were able to assess the impact of this large-scale shift to IC from a macro perspective, which gave us the possibility to follow the population before and after the intervention. Furthermore, having population data made it possible to study the total population and identify sub-groups which might have benefited from IC. The downside to the quasi-experimental approach is our inability to avoid the impact of external influences (24), as we have a long follow-up which coincided with other health and social care reforms which could influence our outcome. Finally, within this study we have no measures of how IC changed how inhabitants experienced care, whether the patients perceived that they received better care or were able to obtain the right combination of health and

For peer review only - http://bmjopen.bmj.com/site/about/guidelines.xhtml

social care services to meet their individual needs. Previous research has observed that healthcare professionals working in an integrated care system perceive the quality of their provided care to be better (22, 30).

Conclusion

In summary, we found that the establishment of the IC initiative in Norrtälje was associated with a modest decrease in the trend of the rate of ED visits among the inhabitants of Norrtälje. Due to the gradual implementation of different aspects of IC (e.g. staff training and shared information system) along with other policy changes in health and social care in the post-intervention period, it was difficult to disentangle the effect of the IC from the other policy changes. Further research is needed to to better understand what impact IC could have on the utilisation of other health and social care services.

For peer review only

For peer review only - http://bmjopen.bmj.com/site/about/guidelines.xhtml

Abbreviations

Emergency Department (ED), Integrated Care (IC), Primary Health Care (PHC), Generalised Linear Model (GLM), Confidence Interval (CI), Incidence Rate Ratio (IRR), Chronic Obstructive Pulmonary Disease (COPD)

Acknowledgements

A special thanks to the Equity and Health Policy research group at the department of Global Public Health at Karolinska Institutet and the Aging Research Center for helpful comments and suggestion of this paper.

Author contributions

MD, JA, NO, PS and BB conceived of the idea for, and participated in the design and the coordination this study. MD performed the statistical analysis and drafted the manuscript. MD, JA, NO, PS and BB were all involved in the interpretation of results and the revisions to the manuscript drafts. All authors read and approved the manuscript.

Funding

The research leading to these results was carried out as part of the Social Inequalities in Ageing (SIA) project, funded by NordForsk, project no.74637 and the Integrated Care Project funded by the Swedish Research Council for Health, Working Life and Welfare (Forte) project no 2017-02155.The funders of this research have had no direct role in the design, data collection, analysis or interpretation of data and in the writing of the manuscript.

Ethical approvals

Ethical approval was obtained for the use of population-based data. The research team applied for, and was granted ethical approval from the Regional Ethical Review Board in Stockholm, Sweden Dnr: 2016/299–31.

Participants consent

Individual consent to participate in this study was waived as this was a population-based study using administrative data.

Data statement

The data used will not be shared, as the materials are based on individual data which cannot be shared in accordance with Swedish law and GDPR.

Competing Interests

The authors declare no competing interests.

References

1. Statistics Sweden: Largest population increase expected among the elderly (2018). https://www.scb.se/en/finding-statistics/statistics-by-subject-area/population/population-

For peer review only

projections/population-projections/pong/statistical-news/the-future-population-of-sweden-2018-2070/
2. WHO, World report on ageing and health: GenevaÖ Workd Health Organisation. 2015.
3. Meinow, B., M.G. Parker, and M. Thorslund, Complex health problems and mortality among the oldest old in Sweden: decreased risk for men between 1992 and 2002. European Journal of Ageing, 2010. 7(2): p. 81-90.
4. Briggs AM, Valentijn PP, Thiyagarajan JA, Araujo de Carvalho I. Elements of integrated care approaches for older people: a review of reviews. BMJ Open. 2018;8(4):e021194
5. Goodwin N. How should integrated care address the challenge of people with complex health and social care needs? Emerging lessons from international case studies. Int J Integr Care, (2015); 15:12.
6. Fritzell J, Salen L, Nelson K, Palme J and Schön P. European Social Policy Network [ESPN[, Country Profile: Sweden. Chapter 2 Systems and policies affecting social outcomes, 2016,2943.
7. Burström, B. (2015). Sweden – recent changes in welfare state arrangements. *International Journal of Health Services, 45*(1), 87-104.
8. Burström, B., Burström, K., Nilsson, G., Tomson, G., Whitehead, M., & Winblad, U. (2017). Equity aspects of the Primary Health Care Choice Reform in Sweden–a scoping review. *International journal for equity in health, 16*(1), 29.
9. SBU, *Omhändertagande av äldre som inkommer akut till sjukhus – med fokus på sköra äldre. En systematisk litteraturöversikt*. 2013: Stockholm: Statens beredning för medicinsk utvärdering [Swedish Council on Health Technology Assessment].
10. Meinow B, Parker M, Thorslund M. Consumers of eldercare in Sweden: The semblance ofchoice. Social Science and Medicine 2011;73;18 p233-248.
11. McCusker, J., Karp, I., Cardin, S., Durand, P., & Morin, J. (2003). Determinants of emergency department visits by older adults: A systematic review. *Academic Emergency Medicine, 10,* 1362-1370.
12. Nugus P, Carroll K, Hewett DG, Short A, Forero R, Braithwaite J. Integrated care in the emergency department: A complex adaptive systems perspective. Social Science and Medicine. 2010 Dec;71(11):1997-2004.
13. Hujala A, Taskinen H RS. How to support integration to promote care for people with multimorbidity in Europe? . Berlin: ICARE-4EU; 2016.
14. Kodner DL., Spreeuwenberg C. (2002) Integrated care: meaning, logic, applications, and implications – a discussion paper, International Journal of Integrated Care, 2, 1-6.
15. Mason A, Goddard M, Weatherly H, Chalkley M. Integrating funds for health and social care: an evidence review. J Health Serv Res Policy. 2015;20(3):177-88.
16. Leutz W. (1999) Five laws for integrating medical and social services: Lessons from the United States and the United Kingdom, Millbank quarterly 77(1), 77-110.
17. Goddard M., Mason AR. (2017) Integrated Care: A Pill for All Ills? International Journal of Health Policy and Management 6(1), 1-3.
18. Singer, S. J., Burgers, J., Friedberg, M., Rosenthal, M. B., Leape, L. L., & Schneider, E. C. (2011). Defining and measuring integrated patient care: Promoting the next frontier in healthcare delivery. *Medical Care Research and Review*, *68*(1), 112-127.
19. Tricco AC, Antony J, Ivers NM, Ashoor HM, Khan PA, Blondal E, et al. Effectiveness of quality improvement strategies for coordination of care to reduce use of health care services: a systematic review and meta-analysis. CMAJ. 2014;186(15):E568-78.
20. Bäck MA, Calltorp J. The Norrtaelje model: a unique model for integrated health and social care in Sweden. Int J Integr Care. 2015;15:e016.

For peer review only - http://bmjopen.bmj.com/site/about/guidelines.xhtml

21. Schön, P., Hagman, L. & Wånell, S.E. (2012). Kan två bli en? TioHundraprojektets effekter på vård och omsorg för målgruppen de mest sjuka äldre. [Can two become one? Implementing an integrated health and social care organization for the oldest old with complex health problems] Rapport 2012:01. Stiftelsen Stockholms läns Äldrecentrum [Stockholm Gerontology Research Center].
22. Øvretveit J, Hansson J, Brommels M. An integrated health and social care organisation in Sweden: creation and structure of a unique local public health and social care system. Health Policy. 2010;97(2-3):113-21.
23. National Board of Health and Welfare [NBHW]. (2018) Dementia strategy focusing on care, status report 2018. Swedeish Socialstyrelsen.
24. Craig P, Katikireddi SV, Leyland A, Popham F. Natural Experiments: An Overview of Methods, Approaches, and Contributions to Public Health Intervention Research. Annu Rev Public Health. 2017;38:39-56.
25. Linden A, Arbor A, Conducting interrupted time-series analysis for single- and multiple-group comparisons. The Stata Journal (2015) 15, Number 2, p 480–500.
26. Lopez Bernal J, Cummins S, A. G. Interrupted time series regression for the evaluation of public health interventions: a tutorial. International Journal of Epidemiology. 2017;10(1093):348-55.
27. Kontopantelis, E., Doran, T., Springate, D. A., Buchan, I., & Reeves, D. (2015). Regression based quasi-experimental approach when randomisation is not an option: interrupted time series analysis. *BMJ (Clinical research ed.)*, *350*, h2750. doi:10.1136/bmj.h2750
28. Ahgren B. Competition and integration in Swedish health care. Health Policy. 2010;96(2):91-7.
29. Leichsenring K, Rodrigues R, Winkelmann J, Falk R. Integrated Care, Competition and Choice - Removing barriers to care coordination in the context of market-oriented governance in Germany and Sweden. International Journal of Integrated Care. 2016;16(6):A89. DOI: http://doi.org/10.5334/ijic.2637
30. Ström Falk R, Strano A & Schön P. Competing logics? The Coextistence of Integrated Care and Customer-choice in the Organisation and Provision of Home-based Care. 2015.
31. Cameron A, Lart R, Bostock L, Coomber C. Factors that promote and hinder joint and integrated working between health and social care services: a review of research literature. Health Soc Care Community. 2014;22(3):225-33.
32. Schiøtz M, Price M, Frølich A, Søgaard J, Kristensen JK, Krasnik A, et al. Something is amiss in Denmark: a comparison of preventable hospitalisations and readmissions for chronic medical conditions in the Danish Healthcare system and Kaiser Permanente. BMC Health Serv Res. 2011;11:347.
33. RAND Europe, Ernst & Young LLP. National evaluation of the Departments of Health's Integrated Care Pilots: Final report. Cambridge. RAND Corporation, 2012.
34. Stephenson MD, Lisy K, Stern CJ, Feyer AM, Fisher L, Aromataris EC. The impact of integrated care for people with chronic conditions on hospital and emergency department utilization: a rapid review. Int J Evid Based Healthc. 2018.
35. Lemmens KM, Lemmens LC, Boom JH, Drewes HW, Meeuwissen JA, Steuten LM, et al. Chronic care management for patients with COPD: a critical review of available evidence. J Eval Clin Pract. 2013;19(5):734-52

For peer review only

Table 1: The trend changes in the rate of ED visits associated with the implementation of integrated care in Norrtälje between 2000-2015

	Rate of ED visits per quarter per 10,000 population		Age adjusted Rate of ED visits per quarter per 10,000 population		Level change (immediately following the intervention)		Trend change (after the intervention)	
	Before	After	Before	After	IRR (CI 95%)	p-value	IRR (CI 95%)	p-value
All inhabitants	1503.5	1562.7	1478.84	1584.17	0.996 (0.971 to 1.022)	0.793	0.997 (0.995 to 0.998)	0.000
Males	1627.0	1620.4	1674.5	1728.0	0.959 (0.937 to 0.983)	0.001	0.997 (0.995 to 0.998)	0.000
Females	1499.5	1510.6	1336.6	1493.6	1.036 (1.009 to 1.064)	0.008	0.995 (0.994 to 0.998)	0.000
65-79 years	1218.1	1287.5	822.7	891.5	1.037 (1.003 to 1.073)	0.034	0.995 (0.993 to 0.997)	0.000
80+ years	2145.8	2298.3	660.05	700.57	0.946 (0.911 to 0.983)	0.005	0.998 (0.996 to 1.00)	0.323
Lowest income	1582.2	1593.0	1449.6	1553.1	0.941 (0.917 to 0.965)	0.000	0.996 (0.994 to 0.997)	0.000
Living alone	1650.3	1764.4	1538.2	1676.6	0.987 (0.963 to 1.011)	0.301	0.996 (0.995 to 0.998)	0.000
Born outside of Sweden	1452.4	1683.0	1591.5	1748.0	0.872 (0.852 to 0.894)	0.000	0.991 (0.989 to 0.992)	0.000

- IRR incidence rate ratio, CI confidence interval
- The step change the level change immediately after the implementation of IC
- The trend change in the rate of ED visits in the post-intervention period

For peer review only - http://bmjopen.bmj.com/site/about/guidelines.xhtml

Figure 1: Overview of the timeline of the implementation of IC in Norrtälje

Figure 2: The trend in the rate of ED visits among all inhabitants 65+ years in Norrtälje from 2000q1 to 2015q4. The circles represent the observed rate of ED visits per 10,000 persons, the vertical line represents the implementation of integrated care (post-intervention trend from 2006q1) The solid line represents the temporal trend, and the step at the postintervention period is the estimated effect and the dashed red line represents the counterfactual trend if integrated care was not implemented in Norrtälje.

Figure 3: The trend in the rate of ED visits among the inhabitants 65+ years in the rest of Stockholm County from 2000q1 to 2015q4. The circles represent the observed rate of ED visits per 10,000 persons, the vertical line represents the point where IC was implemented in Norrtälje. The solid line represents the temporal trend, and the step at the post-intervention period is the estimated effect and the dashed red line represents the counterfactual trend if integrated care was not implemented in Norrtälje.

For peer review only - http://bmjopen.bmj.com/site/about/guidelines.xhtml

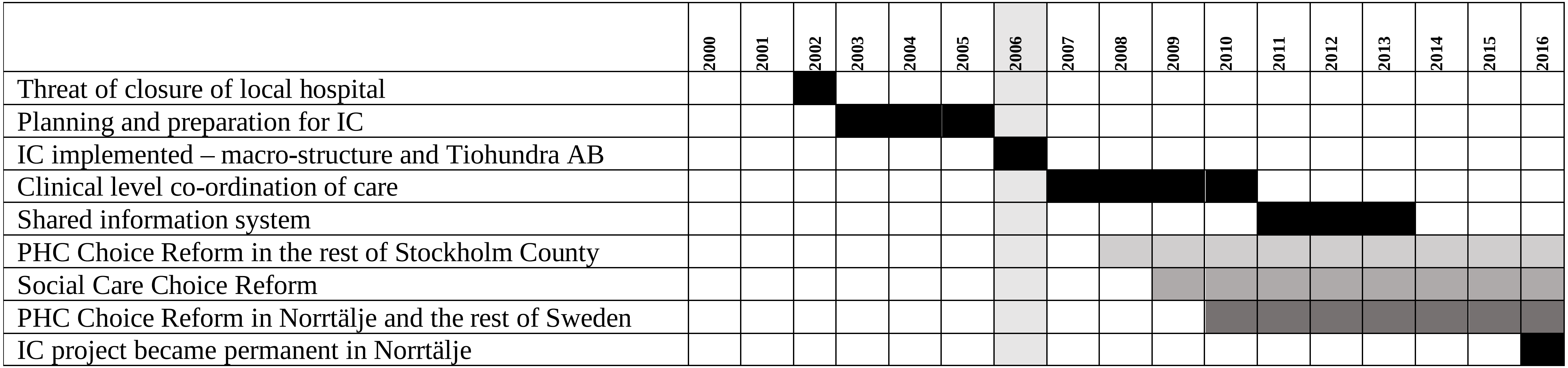
2000
2001
2002
2003
2004
2005
2006
2007
2008
2009
2010
2011
2012
2013
2014
2015
2016
Threat of closure of local hospital
Planning and preparation for IC
IC implemented – macro-structure and Tiohundra AB
Clinical level co-ordination of care
Shared information system
PHC Choice Reform in the rest of Stockholm County
Social Care Choice Reform
PHC Choice Reform in Norrtälje and the rest of Sweden
IC project became permanent in Norrtälje

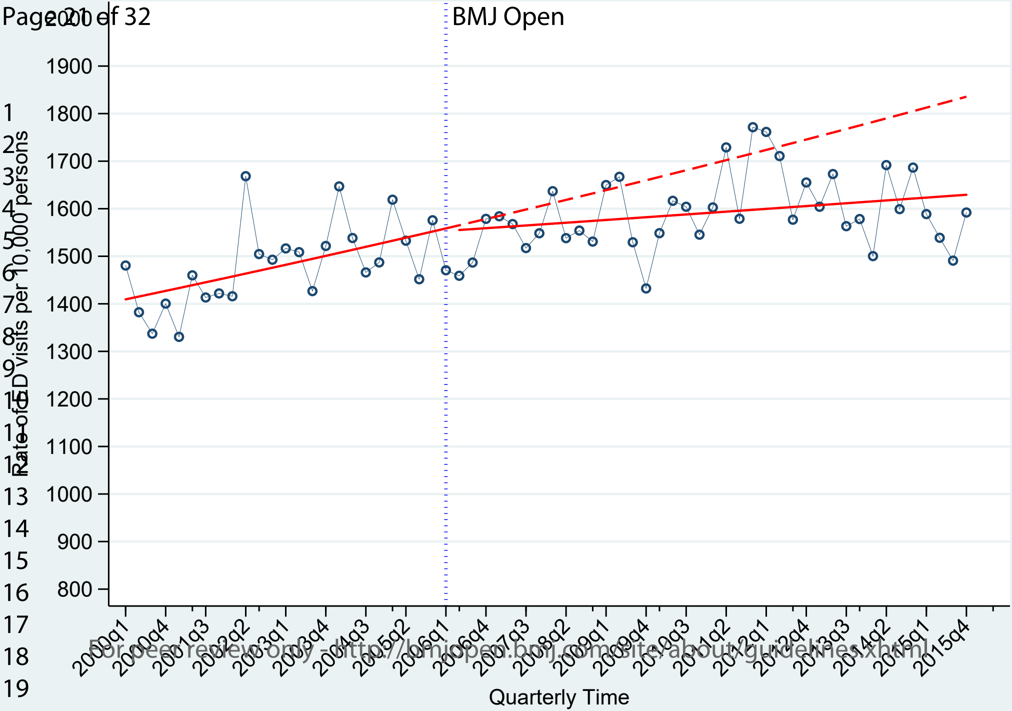

For peer review only - http://bmjopen.bmj.com/site/about/guidelines.xhtml

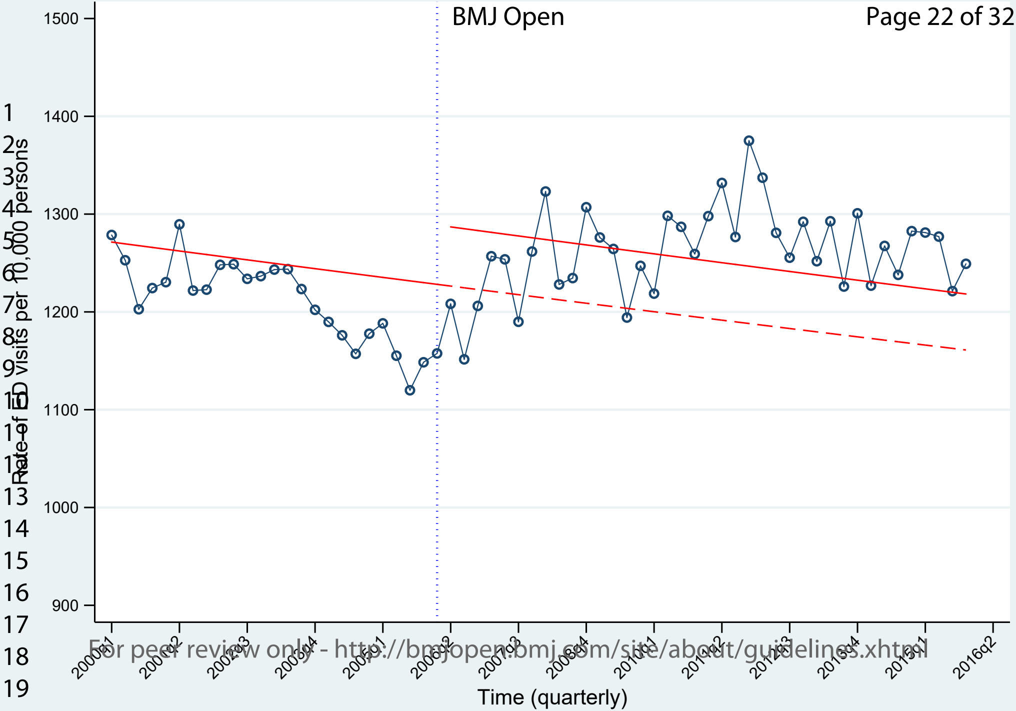

For peer review only - http://bmjopen.bmj.com/site/about/guidelines.xhtml

Supplementary Data

Table S1: Socio-demographic description of the inhabitants of Norrtälje in proportions (%) and means from 2000-2015

	**Year**	**2000**	**2001**	**2002**	**2003**	**2004**	**2005**	**2006**	**2007**	**2008**	**2009**	**2010**	**2011**	**2012**	**2013**	**2014**	**2015**
	N=	10,249	10,460	10,660	10,801	10,921	11,114	11,330	11,704	12,242	12,832	13,103	13,893	14,580	15,039	15,481	16,011
**Variables**		(%)	(%)	(%)	(%)	(%)	(%)	(%)	(%)	(%)	(%)	(%)	(%)	(%)	(%)	(%)	(%)
**Sex**	Male	45.29	45.38	45.54	45.72	45.83	46.07	46.44	46.69	46.80	47.11	47.15	47.47	47.79	48.05	48.08	47.98
	Female	54.71	54.62	54.46	54.28	54.17	53.93	53.56	53.31	53.20	52.89	52.85	52.53	52.21	51.95	51.92	52.02
**Age**	Mean in years	75.69	75.7	75.71	75.71	75.69	75.66	75.56	75.43	75.19	74.98	74.75	74.71	74.57	74.61	74.69	74.78
**Age groups**	65–79 years	74.82	73.85	72.74	72.49	72.53	72.70	72.44	73.11	73.89	74.49	75.74	75.83	76.88	77.12	77.24	77.70
	80+ years	25.18	26.15	27.26	27.51	27.47	27.30	27.56	26.89	26.11	25.51	24.26	24.17	23.12	22.88	22.76	22.30
**Income**	Group 1 (low)	31.00	29.75	29.37	35.54	36.59	35.62	31.29	31.64	31.23	30.75	30.95	30.43	29.21	29.29	28.95	28.22
	Group 2	21.98	23.15	22.32	22.07	21.32	21.51	20.56	20.30	19.80	19.54	19.64	20.15	25.33	25.32	25.21	25.14
	Group 3	19.01	18.12	19.32	17.33	17.21	16.72	18.87	19.28	19.84	20.60	20.38	2.50	17.25	17.90	17.74	18.27
	Group 4	14.06	14.56	14.50	14.95	14.74	15.25	16.92	17.06	16.76	16.97	16.96	34.38	15.49	15.07	15.35	15.30
	Group 5 (high)	9.02	9.71	9.55	9.99	10.03	10.80	12.10	11.44	12.18	11.86	11.77	12.27	11.98	11.69	11.99	12.39
	Missing	4.94	4.71	4.94	0.11	0.11	0.10	0.26	0.28	0.19	0.29	0.31	0.26	0.75	0.72	0.75	0.67
**Country of Birth**	Other	8.76	9.14	9.45	9.65	9.92	10.13	10.61	10.73	10.65	10.50	10.46	10.57	10.58	10.59	10.70	10.66
	Sweden	91.24	90.86	90.55	90.35	90.08	89.87	89.39	89.27	89.35	89.50	89.54	89.43	89.42	89.41	89.30	89.34
**Living situation**	Cohabitating	55.75	51.63	52.14	51.28	54.69	54.67	54.43	54.94	55.61	56.11	56.45	56.55	55.85	55.62	55.61	55.30
	Alone	44.25	43.30	43.02	43.74	45.31	45.33	45.57	45.06	44.39	43.89	43.55	43.45	44.15	44.38	44.39	44.70
	Missing		5.08	4.84	4.98												
	Died	4.84	4.58	4.84	4.98	4.77	4.87	4.57	4.37	4.23	4.11	4.25	4.05	3.61	3.78	3.40	3.49
**ED utilisation**	None	70.29	69.93	68.12	68.93	69.28	68.95	69.22	68.75	67.64	67.99	68.26	67.4	67.52	68.05	69.32	69.13
	1 visits	16.93	17.2	17.9	17.36	16.55	16.54	16.8	16.97	18.1	17.97	17.71	18.4	17.7	17.95	16.36	17.18
	2–3 visits	9.53	9.85	10.49	10.05	10.16	10.5	10.1	10.3	10.52	10.29	10.32	10.22	10.51	10.17	10.24	9.83
	4+ visits	3.25	3.02	3.49	3.67	4.01	4.01	3.87	3.98	3.74	3.76	3.72	3.97	4.27	3.83	4.08	3.85
**ED visits**	Mean no. visits	0.57	0.57	0.62	0.61	0.63	0.63	0.61	0.63	0.63	0.62	0.62	0.65	0.66	0.64	0.64	0.62

Table S2: Socio-demographic description of the inhabitants aged 65+ years of the rest of Stockholm County in proportions (%) and means from 2000-2015

	**Year**	**2000**	**2001**	**2002**	**2003**	**2004**	**2005**	**2006**	**2007**	**2008**	**2009**	**2010**	**2011**	**2012**	**2013**	**2014**	**2015**
	N=	261,608	262,922	262,398	263,688	265,654	268,125	272,192	278,409	286,508	296,660	307,293	316,813	326,014	334,981	343,047	349,958
**Variables**		(%)	(%)	(%)	(%)	(%)	(%)	(%)	(%)	(%)	(%)	(%)	(%)	(%)	(%)	(%)	(%)
**Sex**	Male	40.19	40.41	40.66	40.97	41.27	41.6	41.88	42.25	42.7	43.08	43.44	43.71	43.94	44.15	44.33	44.57
	Female	59.81	59.59	59.34	59.03	58.73	58.4	58.12	57.75	57.3	56.92	56.56	56.29	56.06	55.85	55.67	55.43
**Age**	Mean in years	76.1	76.14	76.18	76.16	76.12	76.09	75.99	75.81	75.58	75.35	75.17	75.02	74.92	74.86	74.88	74.92
**Age groups**	65–79 years	68.06	67.19	66.67	66.41	66.27	66.22	66.62	67.45	68.41	69.56	70.47	71.39	72.24	73.08	73.65	74.08
	80+ years	31.94	32.81	33.33	33.59	33.73	33.78	33.38	32.55	31.59	30.44	29.53	28.61	27.76	26.92	26.35	25.92
**Income**	Group 1 (low)	18.47	18.17	18.5	19.21	19.13	19.14	19.33	19.37	19.37	19.39	19.35	20.03	19.33	19.31	19.33	19.4
	Group 2	18.86	18.9	18.51	19.88	19.93	19.84	19.9	19.9	19.93	19.84	19.96	19.66	19.51	19.47	19.52	19.59
	Group 3	18.97	18.95	19.31	20	20.04	20.15	19.91	19.94	19.89	19.87	19.88	19.87	19.87	19.87	19.85	19.88
	Group 4	19.17	19.27	19.12	20.1	20.12	20.11	20.01	20	20.05	20.01	20.01	20.02	19.94	19.96	19.97	20.03
	Group 5 (high)	19.23	19.29	19.17	20.26	20.26	20.25	20.16	20.19	20.19	20.17	20.21	20.26	20.11	20.12	20.11	20.16
	Missing	5.31	5.43	5.4	0.56	0.52	0.5	0.69	0.6	0.58	0.71	0.6	0.16	1.24	1.26	1.22	0.95
**Country of Birth**	Other	16.53	17.06	17.6	18.06	18.53	18.96	19.35	19.41	19.47	19.48	19.5	19.76	20.09	20.4	20.75	21.19
	Sweden	83.47	82.94	82.4	81.94	81.47	81.04	80.65	80.59	80.53	80.52	80.5	80.24	79.91	79.6	79.25	78.81
**Living situation**	Cohabitating	48.66	45.41	45.76	45.84	48.9	48.93	48.97	49.25	49.64	50.01	50.39	50.7	49.91	50.22	50.38	50.53
	Alone	51.23	49.07	49.31	49.3	51	50.97	50.84	50.57	50.18	49.81	49.45	49.14	50.09	49.78	49.62	49.47
	Missing	0.12	5.53	4.93	4.86	0.1	0.11	0.19	0.19	0.18	0.18	0.16	0.16				
	Died	4.83	4.79	4.93	4.86	4.68	4.69	4.64	4.61	4.42	4.13	4.15	4.04	3.93	3.73	3.77	3.8
**ED utilisation**	None	71.02	70.75	71.29	71.35	72.36	72.1	69.02	67.99	69.02	70.62	70.63	70.24	70.77	71.02	71.55	70.11
	1 visits	15.98	16.41	16.12	16.3	16	16.32	17.66	17.58	17.23	16.7	16.68	16.73	16.57	16.52	16.3	17.22
	2–3 visits	9.77	9.71	9.52	9.38	8.91	8.9	10.13	10.65	10.18	9.56	9.55	9.75	9.52	9.4	9.22	9.55
	4+ visits	3.23	3.13	3.07	2.98	2.73	2.67	3.18	3.78	3.57	3.13	3.14	3.28	3.14	3.06	2.93	3.13
**ED visits**	Mean no. visits	0.56	0.56	0.55	0.54	0.52	0.51	0.59	0.64	0.61	0.56	0.56	0.58	0.56	0.55	0.54	0.57

For peer review only - http://bmjopen.bmj.com/site/about/guidelines.xhtml

Table S3: Description of ED care utilisation among stratified sub-groups in Norrtälje and the rest of Stockholm County.

	Norrtälje		Rest of Stockholm	
	**2000-06**	**2006-15**	**2000-06**	**2006-15**
**All inhabitants**				
**N=**	10,726	13,680.3	264,398.9	312,187.4
**Average no. per quarter**	1614.2	2137.7	33,037.5	39,043.95
**Average no. per quarter per person**	0.1503	0.1562	0.1250	0.1252
**Rate per quarter per 10,000 persons**	1503.5	1562.7	1249.9	1251.5
**Age adjusted rate per quarter per 10,000 persons**	1478.84	1584.17	1210.8	1262.3
**Males**				
**N=**	4,899.6	6,490	108,124.6	135,840.5
**Average no. per quarter**	798.1	1052.1	13,812.8	16,731.2
**Average no. per quarter per person**	0.163	0.162	0.128	0.125
**Rate per quarter per 10,000 persons**	1627.1	1620.4	1278.3	1252.3
**Age adjusted rate per quarter per 10,000 persons**	1674.5	1728.0	1308.7	1338.1
**Females**				
**N=**	5826.4	7,190.3	156,266.3	176.346.8
**Average no. per quarter**	816.1	1085.6	19224.7	22061.8
**Average no. per quarter per person**	0.14	0.15	0.12	0.13
**Rate per quarter per 10,000 persons**	1399.5	1510.6	1230.3	1251.8
**Age adjusted rate per quarter per 10,000 persons**	1336.6	1493.6	1135.2	1217.1
**65-79 years**				
**N=**	7427.7	9957.4	176,590.6	221,647
**Average no. per quarter**	905.2	1280.7	17880.2	21705.8
**Average no. per quarter per person**	0.12	0.13	0.10	0.098
**Rate per quarter per 10,000 persons**	1218.1	1287.5	1012.7	980.9
**Age adjusted rate per quarter per 10,000 persons**	822.7	891.5	681.7	681.8
**80+ years**				
**N=**	3,298.3	3,722.9	87,800.2	90,540.4
**Average no. per quarter**	708.9	857	15157.2	17330.2
**Average no. per quarter per person**	0.22	0.23	0.17	0.19
**Rate per quarter per 10,000 persons**	2145.8	2298.3	1727.0	1915.0
**Age adjusted rate per quarter per 10,000 persons**	660.05	700.57	529.3	580.4
**Lowest income**				
**N=**	3,536	4,060	49,630	60,637.3
**Average no. per quarter**	563.1	646.5	6843.5	7934.1
**Average no. per quarter per person**	0.158	0.159	0.14	0.13
**Rate per quarter per 10,000 persons**	1582.2	1593	1377.8	1307.4
**Age adjusted rate per quarter per 10,000 persons**	1449.6	1553.1	1261.9	1289.5
**Living alone**				
**N=**	4,744.1	6,061.2	132,660.9	155,606.1
**Average no. per quarter**	784.5	1071.2	17902.2	22492.0
**Average no. per quarter per person**	0.17	0.18	0.13	0.15
**Rate per quarter per 10,000 persons**	1650.3	1764.4	1349.2	1445.2
**Age adjusted rate per quarter per 10,000 persons**	1538.2	1676.6	1249.7	1360.9
**Born outside of Sweden**				
**N=**	1,026	1,451	47,216.8	62,437.7
**Average no. per quarter**	150.1	245.4	5679.6	7925.4
**Average no. per quarter per person**	0.15	0.17	0.12	0.13
**Rate per quarter per 10,000 persons**	1452.4	1683.0	1204.1	1268.3
**Age adjusted rate per quarter per 10,000 persons**	1591.5	1748.0	1250.0	1312.8

Supplementary figures corresponding to stratified analysis of socio-demographic sub-groups in Norrtälje municipality, the figures correspond to the estimates present in Table 1 of the main text.

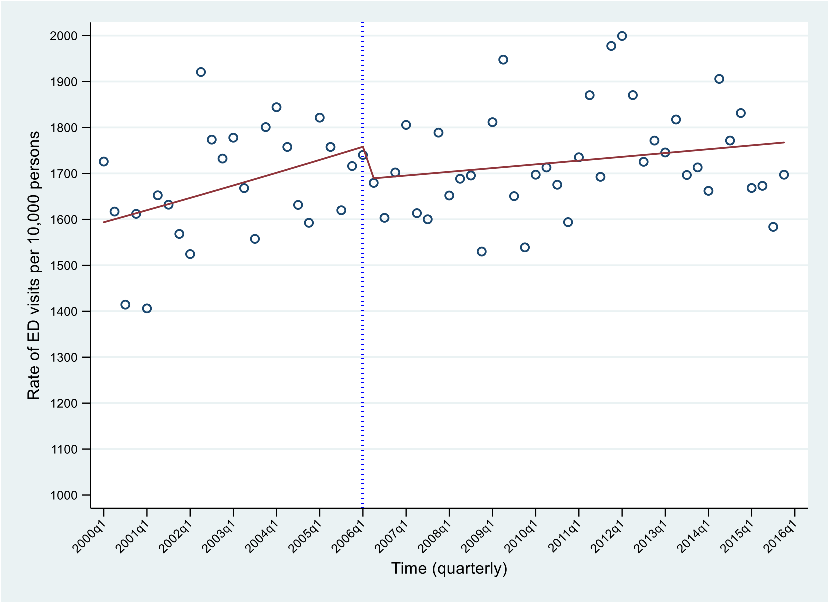

Figure S1: The trend in the rate of ED visits among male inhabitants in Norrtälje from 2000q1 to 2015q4. The circles represent the observed rate of ED visits per quarter per 10,000 persons, the vertical line represents the point where IC was implemented in Norrtälje. The solid lines represent the changes in the trend of the rate of ED visits in the pre-and post-intervention periods.

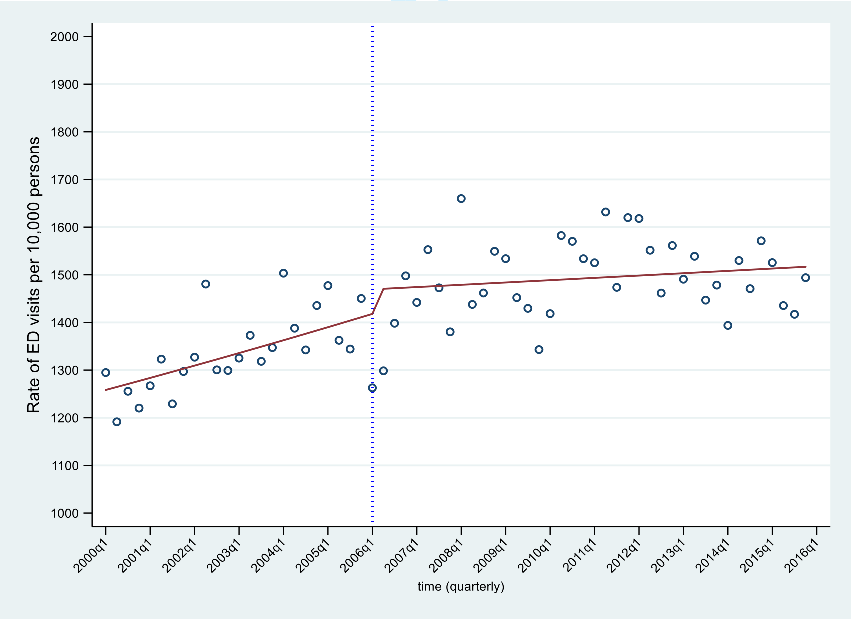

Figure S2: The trend in the rate of ED visits among female inhabitants in Norrtälje from 2000q1 to 2015q4. The circles represent the observed rate of ED visits per quarter per 10,000 persons, the vertical line represents the point where IC was implemented in Norrtälje. The solid lines represent the changes in the trend of the rate of ED visits in the pre-and post-intervention periods.

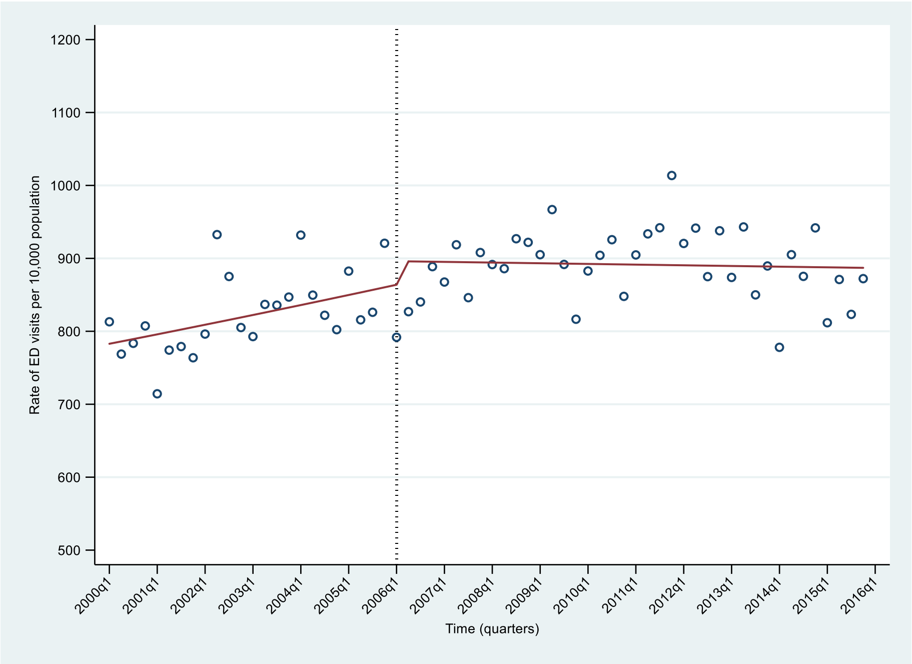

Figure S3: The trend in the rate of ED visits among all those 65-79 years in Norrtälje from 2000q1 to 2015q4. The circles represent the observed rate of ED visits per quarter per 10,000 persons, the vertical line represents the point where IC was implemented in Norrtälje. The solid lines represent the changes in the trend of the rate of ED visits in the pre-and post-intervention periods.

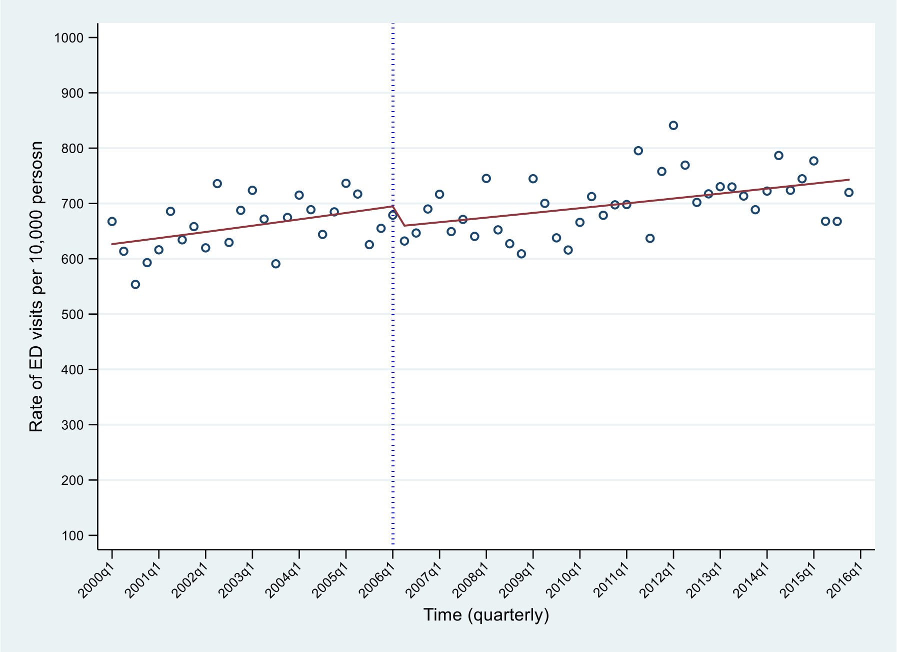

Figure S4: The trend in the rate of ED visits among all those 80+ years in Norrtälje from 2000q1 to 2015q4. The circles represent the observed rate of ED visits per quarter per 10,000 persons, the vertical line represents the point where IC was implemented in Norrtälje. The solid lines represent the changes in the trend of the rate of ED visits in the pre-and post-intervention periods.

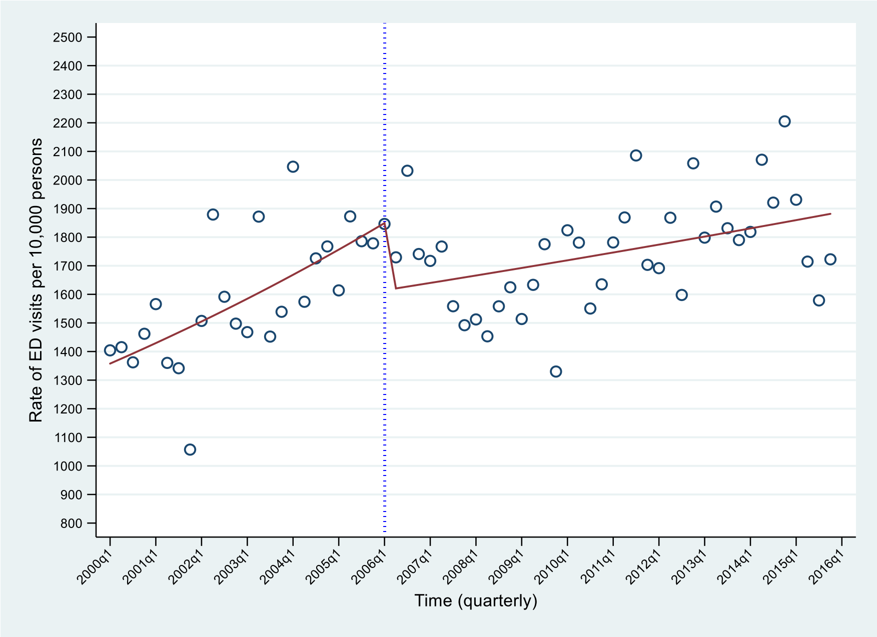

Figure S5: The trend in the rate of ED visits among all those born outside of Sweden living in Norrtälje from 2000q1 to 2015q4. The circles represent the observed rate of ED visits per quarter per 10,000 persons, the vertical line represents the point where IC was implemented in Norrtälje. The solid line represents the smooth trend of the rate of ED visits.

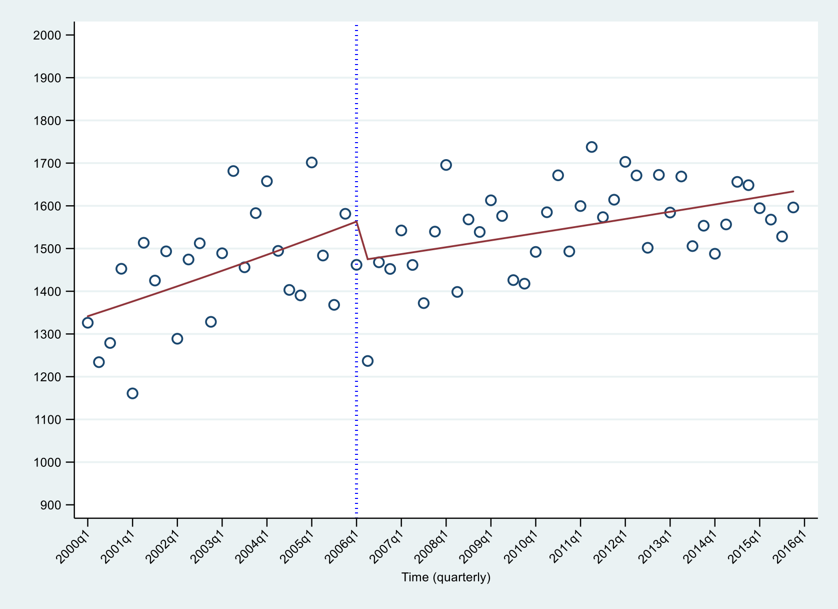

Figure S6: The trend in the rate of ED visits among all those in the lowest income group in Norrtälje from 2000q1 to 2015q4. The circles represent the observed rate of ED visits per quarter per 10,000 persons, the vertical line represents the point where IC was implemented in Norrtälje. The solid line represents the changes in the trend of the rate of ED visits in the pre-and post-intervention periods.

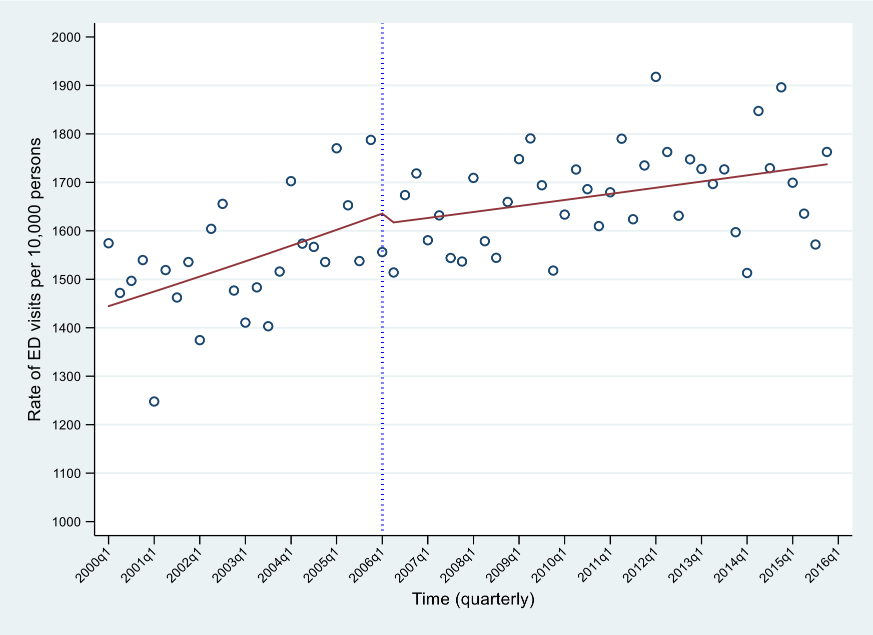

Figure S7: The trend in the rate of ED visits among all those living alone in Norrtälje from 2000q1 to 2015q4. The circles represent the observed rate of ED visits per quarter per 10,000 persons, the vertical line represents the point where IC was implemented in Norrtälje. The solid lines represent the changes in the trend of the rate of ED visits in the pre-and post-intervention periods.

For peer review only

Sensitivity Analysis

In the pre-intervention period 2000q1 to 2005q4 the trend of the rate of ED visits was increasing, following the implementation of IC, there was no significant level change (IRR: 0.971 CI 0.908 to 1.038), followed by a moderately decreasing change in the trend (IRR: O.999 CI: 0.996 to 1.01) in the post-intervention period relative to the pre-intervention period, however, this trend change was not significant. Though the changes in the rate of ED visits were not significant during the shortened study period 2000-2008. The trend follows the same direction as the observed in the main text figure 2, but the change was not as strong with the shorter follow-up period 2000-2008.

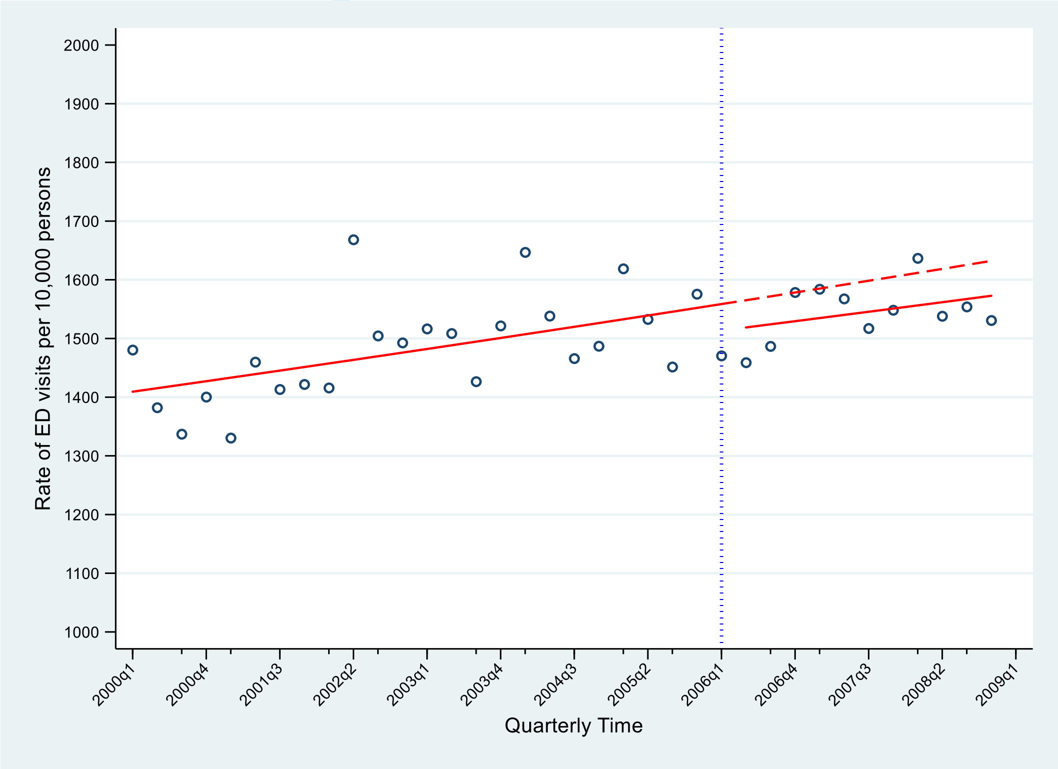

Figure S8: The trend in the rate of ED visits among all inhabitants 65+ years in Norrtälje from 2000q1 to 2008q4. The circles represent the observed rate of ED visits per 10,000 persons, the vertical line represents the implementation of integrated care (post-intervention trend from 2006q1) The solid line represents the temporal trend, and the step at the postintervention period is the estimated effect and the dashed red line represents the counterfactual trend if integrated care was not implemented in Norrtälje.

STROBE Statement—Checklist of items that should be included in reports of ***cross-sectional/cohort studies***

	Item No	Recommendation
**Title and abstract**	1	(*a*) Indicate the study's design with a commonly used term in the title or the abstract<br>Yes the title indicates the study design and includes commonly used terms
		(*b*) Provide in the abstract an informative and balanced summary of what was done and what was found<br>Yes, the abstract gives an informative summary of the what was done and the results of the study
**Introduction**		
Background/rationale	2	Explain the scientific background and rationale for the investigation being reported<br>In the manuscript pages 3 – 5 explain the scientific background and rationale for the study, the ageing population, the Swedish health and social care system, the implications for emergency care use, what is integrated care theoretically and the Norrtalje Model of integrated care.
Objectives	3	State specific objectives, including any prespecified hypotheses<br>The aim of the study is outlined on page 5.
**Methods**		
Study design	4	Present key elements of study design early in the paper<br>The key aspects of the study design and population are outlined in the methods section page 5 – 6.
Setting	5	Describe the setting, locations, and relevant dates, including periods of recruitment, exposure, follow-up, and data collection<br>The relevant dates and outline of the study period and exposure and described on page 5, and in figure 1. The description of data sources is outline on page 6.
Participants	6	(*a*) Give the eligibility criteria, and the sources and methods of selection of participants<br>In methods section page 6.
Variables	7	Clearly define all outcomes, exposures, predictors, potential confounders, and effect modifiers. Give diagnostic criteria, if applicable<br>The outcome of ED visit is defined in the in the Data Sources in the Methods section, and further detail on how it was handled for the analysis is given in the Statistical Analysis section.
Data sources/ measurement	8*	For each variable of interest, give sources of data and details of methods of assessment (measurement). Describe comparability of assessment methods if there is more than one group<br>Each variable included in the time series analysis was described on page 7.
Bias	9	Describe any efforts to address potential sources of bias<br>Efforts were taken to account for changes in the composition of the study population over time, by calculating age standardised rate of ED visits and accounting for changes in trends over time.
Study size	10	Explain how the study size was arrived at<br>The study included all those 65\+ years who were registered as living in Stockholm County on the 31st of December at each year of the study period 2000-2015, if an inhabitants died during that year they were still counted in the study population, if an inhabitant moved out of Stockholm Country during the study period they were excluded from the study population.

For peer review only - http://bmjopen.bmj.com/site/about/guidelines.xhtml

Quantitative variables	11	Explain how quantitative variables were handled in the analyses. If applicable, describe which groupings were chosen and why<br>Description of how the quantitative variables were handled is described in the Statistical Analysis section.
Statistical methods	12	(*a*) Describe all statistical methods, including those used to control for confounding
		(*b*) Describe any methods used to examine subgroups and interactions<br>Stratified sub-groups and interactions included in the analysis were described in the Statistical Analysis section.
		(*c*) Explain how missing data were addressed<br>There was no missing data applicable over study period
		(*d*) If applicable, describe analytical methods taking account of sampling strategy
		(*e*) Describe any sensitivity analyses<br>None
**Results**		
Participants	13*	(a) Report numbers of individuals at each stage of study—eg numbers potentially eligible, examined for eligibility, confirmed eligible, included in the study, completing follow-up, and analysed<br>The number of inhabitants in Norrtalej and the rest of Stockholm are reported in the main text and in the supplementary data table 1 and table 2.
		(b) Give reasons for non-participation at each stage<br>None
		(c) Consider use of a flow diagram<br>None
Descriptive data	14*	(a) Give characteristics of study participants (eg demographic, clinical, social) and information on exposures and potential confounders<br>The characteristics of the inhabitants of Norrtalje during the time period and the inhabitants of the rest of Stockholm are available in the supplementary data table 1 and table 2.
		(b) Indicate number of participants with missing data for each variable of interest<br>None
Outcome data	15*	Report numbers of outcome events or summary measures<br>Are available in table 1 of the main text and in supplementary data table 3.
Main results	16	(*a*) Give unadjusted estimates and, if applicable, confounder-adjusted estimates and their precision (eg, 95% confidence interval). Make clear which confounders were adjusted for and why they were included
		(*b*) Report category boundaries when continuous variables were categorized
		(*c*) If relevant, consider translating estimates of relative risk into absolute risk for a meaningful time period
Other analyses	17	Report other analyses done—eg analyses of subgroups and interactions, and sensitivity analyses<br>Additional analysis is presented in the results section and in the supplementary data.
**Discussion**		
Key results	18	Summarise key results with reference to study objectives<br>Key findings relating to the study aim are discussed on page 9.
Limitations	19	Discuss limitations of the study, taking into account sources of potential bias or

For peer review only

		imprecision. Discuss both direction and magnitude of any potential bias<br>The key findings are discussed on page 9 -10, in terms of limitations of our findings,
Interpretation	20	Give a cautious overall interpretation of results considering objectives, limitations, multiplicity of analyses, results from similar studies, and other relevant evidence<br>The strengths and limitations of the study are outlined on page 11-12.
Generalisability	21	Discuss the generalisability (external validity) of the study results<br>Is discussed in the Discussion section, under the heading “Strengths and Limitations” on page 12.
**Other information**		
Funding	22	Give the source of funding and the role of the funders for the present study and, if applicable, for the original study on which the present article is based<br>Information on funding is available on page 14, placed before the references

For peer review only
